# Engineering a Mechanoresponsive DNA Origami Capsule for Drug Delivery to Narrowed Arteries

**DOI:** 10.1021/acs.nanolett.5c04066

**Published:** 2026-01-05

**Authors:** Hadas Omer, Hadeel Khamis, Zipora Lansky, Racheli Boeangiu, Netanel Korin, Ariel Kaplan, Yuval Garini

**Affiliations:** † Faculty of Biomedical Engineering, 26747 Technion—Israel Institute of Technology, Haifa 3200003, Israel; ‡ Russell Berrie Nanotechnology Institute, Technion—Israel Institute of Technology, Haifa 3200003, Israel; § Faculty of Biology, Technion—Israel Institute of Technology, Haifa 3200003, Israel; ∥ Faculty of Physics, Technion—Israel Institute of Technology, Haifa 3200003, Israel; ⊥ Department of Chemical Engineering, Technion − Israel Institute of Technology, Haifa 3200003, Israel

**Keywords:** DNA origami, self-assembly, AFM, cryo-TEM, shear stress, optical tweezers, polymer, vascular stenosis, drug delivery

## Abstract

Since their inception, DNA origami nanostructures (DONs) have attracted great interest for their programmable geometry, nanoscale precision, and biocompatibility. Here, we present mechanoresponsive DONs designed for targeted drug delivery to narrowed or obstructed arteries. Unlike conventional systems triggered by biochemical cues or external stimuli, our capsules respond autonomously to elevated local shear forces characteristic of stenotic blood flow. The design consists of a hollow boxlike structure sealed by two lids connected through flexible DNA springs, enabling mechanical opening under pathological flow conditions. The nanostructures were assembled and characterized by atomic force microscopy and cryo-transmission electron microscopy, and the mechanical response of the DNA springs was evaluated using optical tweezers. The results confirm that the capsules can operate within physiologically relevant force ranges, demonstrating their potential for noninvasive, site-specific drug release. This mechanoresponsive strategy offers a new paradigm for smart, force-activated nanocarriers for targeted diagnosis and therapy.

## Graphical abstract

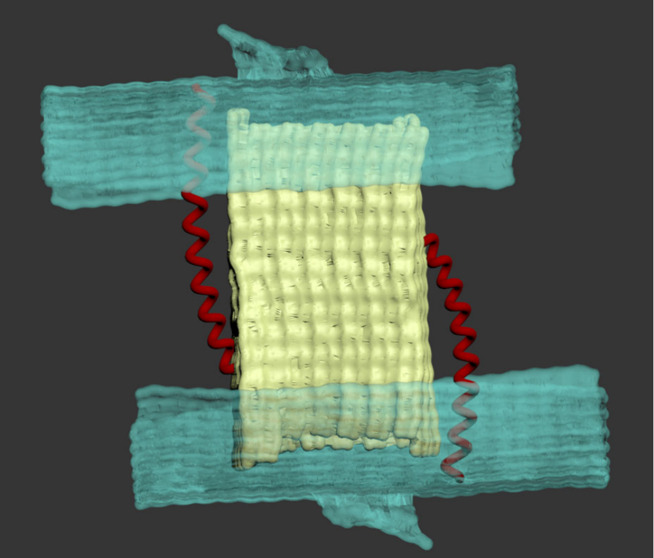

Structural DNA nanotechnology, pioneered by Seeman in the 1980s,^1^ laid the foundation for the DNA origami method introduced by Rothemund in 2006,^2^ in which a long single-stranded DNA (ssDNA) scaffold folds into precise 2D and 3D structures using a set of shorter complementary “staple” strands. DNA origami nanostructures (DONs) offer nanometer-scale precision, biocompatibility, and tunable biochemical and biophysical properties, making them attractive platforms for drug delivery.^3^

Over the past decade, DNA origami has seen extensive development across a variety of biomedical applications, including drug delivery for cancer treatment,^4^ viral trapping,^5^ and immunotherapy,^6^ to name but a few.

A number of studies have investigated DONs for thrombosis,^7^ where arterial obstruction can lead to life-threatening conditions such as myocardial infarction, stroke and pulmonary embolism. Although tissue plasminogen activator (tPA) is an effective clot-dissolving drug, its use is limited by severe off-target effects, especially intracranial bleeding.^8^ Previous approaches for targeted drug delivery in thrombolysis include nanostructures^9^ or platelet-mimicking polymeric nanoparticles,^8^ and a few explored the potential of DNA origami.^10,11^ However, these approaches rely on molecular or enzymatic markers^11–13^ that may also appear in benign plaques, thus lacking the specificity needed to distinguish pathological from benign lesions.

Here we present an origami-based approach for targeted drug delivery to narrowed blood vessels (Figure 1), which exploits a physical cue: the elevated shear force present in pathological vessel constriction. Shear stress is low in healthy vessels but rises sharply in obstructed regions,^14^ exceeding levels found even in the smallest capillaries. These mechanical conditions can be harnessed to achieve spatially precise drug release with reduced off-target effects^9,15^ (Figure 1C,D).

**Figure 1:**
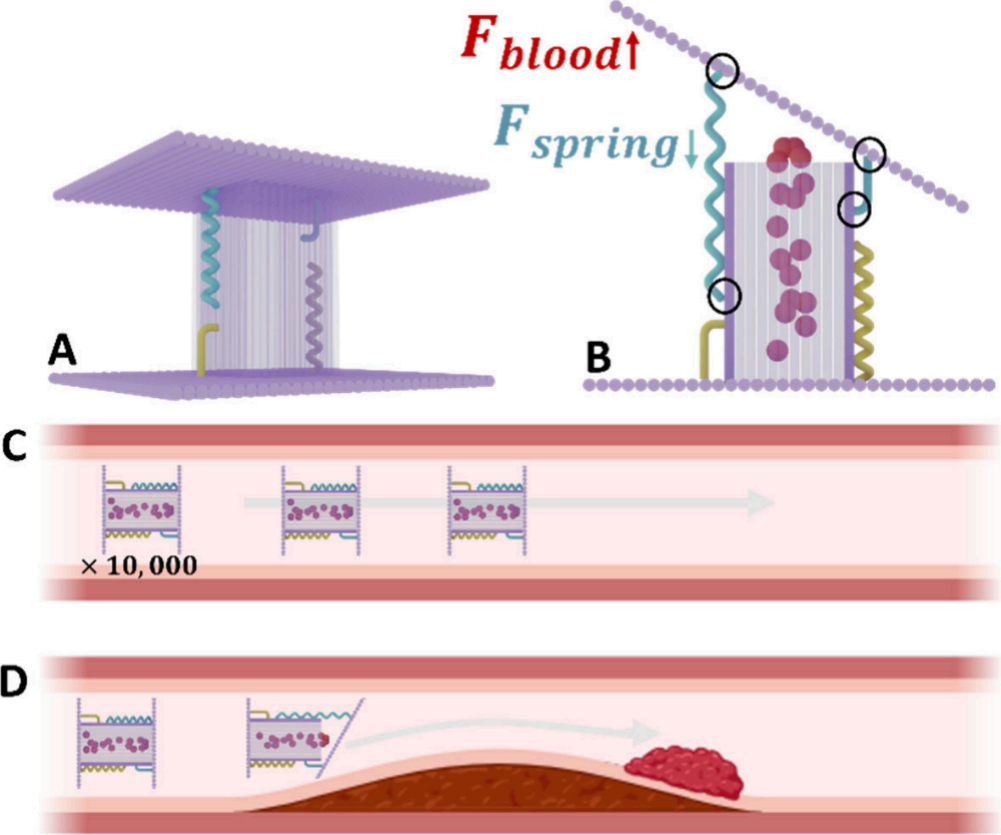
Principle behind mechanoresponsive DNA origami structures for targeted drug delivery to narrowed blood vessels. It relies on shear forces generated in narrowed blood vessels that can stretch the DNA-based spring, causing the capsule to open and release its therapeutic load. (A) Schematic of the DNA origami structure. It consists of a hollow capsule enclosed by two large lids connected to the capsule via hinges and springs. (B) Elevated shear forces stretch a DNA-based spring and open the capsule to release its therapeutic load. Black circles indicate attachment points between the capsule and the upper lid. For a more detailed physical description, see Supplementary Material section 7. (C) Under normal physiological flow, shear forces are low, and the spring elasticity ensures that the capsule stays closed. (D) In a narrowed vessel, the shear force increases significantly and triggers the opening of the capsule, thereby releasing the drug to the blood clot. Note: Capsules are not to scale, they are ∼10^4^ times smaller than illustrated. Partially created with BioRender.

It should be emphasized that elevated shear stress and disturbed flow occur only in narrowed arteries and are absent in healthy vessels. In contrast, molecular or enzymatic markers can also appear in benign plaques that do not obstruct flow, risking drug delivery to unintended sites and potential vessel damage. Thus, mechanical cues offer a level of specificity that molecular triggers alone cannot guarantee.

Although mechanoresponsive DONs have been developed for various applications,^16^ mechanical forces have been employed primarily for exploring the DNA origami itself^17,18^ or for single-molecules studies of attached complexes, such as for exploring the stability and interactions of nucleosomes.^19,20^

To address the challenge of drug delivery to sites of narrowed or obstructed arteries, we designed and synthesized a DNA origami capsule (DOC) composed of a hollow box, capable of carrying drug formulations such as tPA. The box is enclosed from two opposing sides by larger rectangular lids (for the design, see Figures s1–s4 and Tables s1–s14). Each lid is connected to the box by hinges at one edge and a DNA spring at the opposite one (Figures 1B and 2A). The DNA spring is designed to keep the lids closed under normal blood flow and open only when elevated shear forces are present in narrowed vessels.

**Figure 2:**
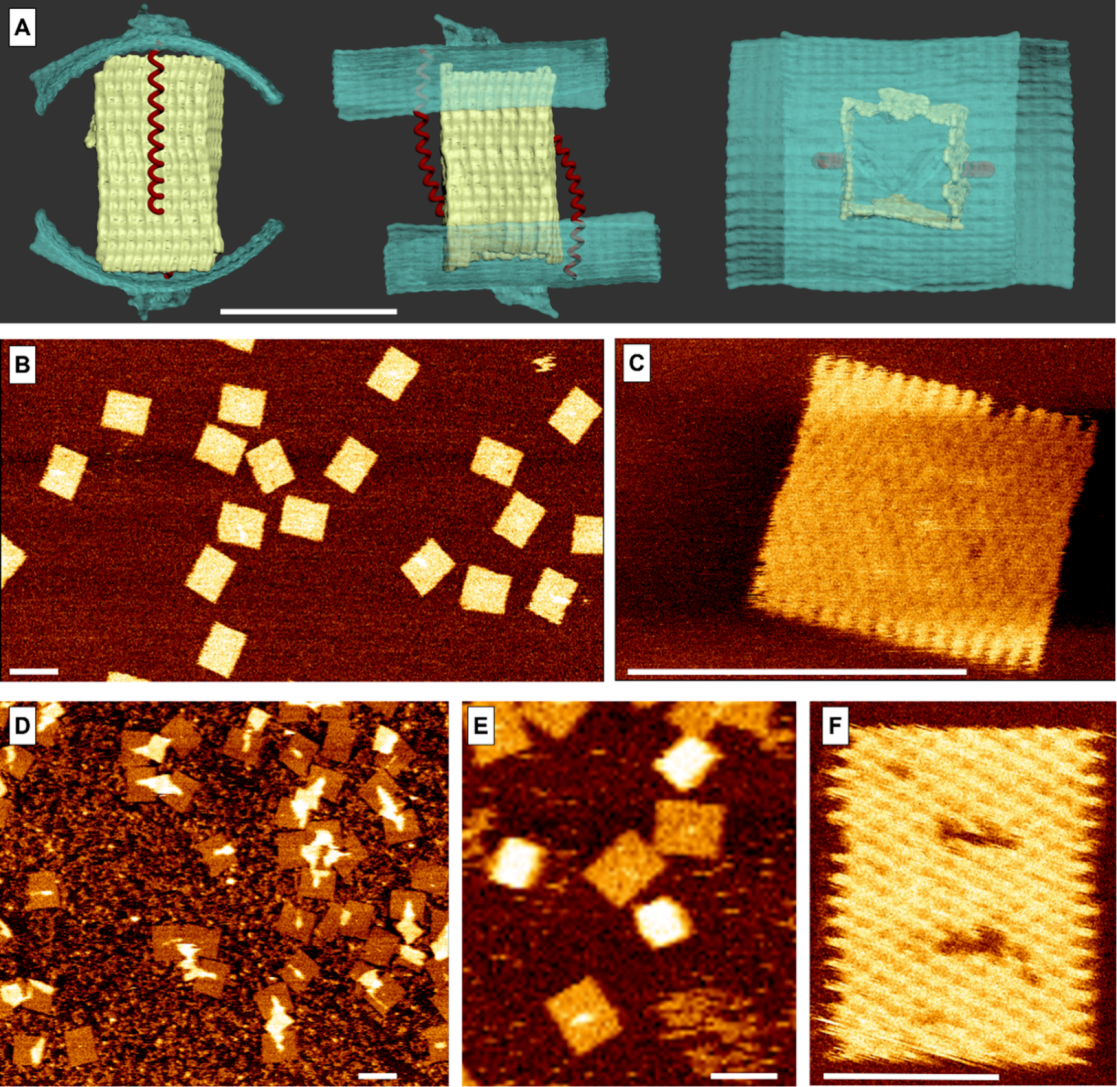
DOC design and its AFM measurements. (A) SNUPI-based^24^ simulation () of the DOC, showing a hollow capsule (yellow) sealed by two opposing lids (turquoise). Each lid is connected by a hinge on one edge and a spring that pulls it toward the capsule on the other (red, not to scale). The small bumps on the lid’s surface are made from a short extra tail that remains from the scaffold after the design. Scale bar = 50 nm. (B and C) AFM images of the lids demonstrate high yield and a structure that aligns with our design. Scale bar = 100 nm. (D and E) AFM images of the box with both lids. Scale bar = 100 nm. The boxes are thicker, and they adhere to the surface so that its 3D structure cannot be analyzed. Some capsules are stretched while the lids adhere to the surface. (F) Lid with designed holes to demonstrate the position and size of the box. Scale bar = 50 nm. See for the missing staples.

Another version of the construction uses only short DNA hinges (DOCHs) to keep the lids closed. Here, the opening mechanism relies on the disruption of multiple hybridization of relative short double-stranded DNA (dsDNA) segments, which may open via unzipping or shearing modes of hybridization.^21,22^

The boxes and lids were initially designed using caDNAno^23^ and their shape and curvature, which can arise from intrinsic mechanical stress, were examined using SNUPI software^24^ (Figure 2A). The structures were subsequently refined through several iterations, in which selected staples were shortened (Figure S1) to relieve tension and improve planarity,^24^ resulting in sufficiently flat lids that ensure full closure of the capsule. The dimensions of the boxes were designed to be 32.5 × 32.5 × 50 nm^3^ to accommodate at least 10 drug molecules. If necessary, larger structures can be synthesized to increase payload capacity. The lids were designed with a rectangular geometry and dimensions (76 × 90 nm^2^) that exceed the capsule’s cross-section, enabling the shear force from blood flow to act over a broad surface.

To visualize the structures, we used atomic force microscopy (AFM) in peak-force tapping mode. Parts B and C of Figure 2 show the lids, and parts D and E of Figure 2 show the boxes with lids (see also Figures s5–s8). The lids are rather flat while the boxes are thicker, but their 3D structure cannot be discerned as they tend to adhere to the mica surface. Figure 2F shows a special structure we synthesized to demonstrate the size difference between the box and the lid, where the holes in the lid mark the size of the box. Overall, the AFM data confirms the structure architecture and high assembly yield (Figure 2B,D).

To assess the three-dimensional shape and scale of the DOC, we used cryo-TEM (Figure 3); for details, see Supplementary Material section 5. Three sets of samples were prepared: (1) boxes only (Figures 3A–C and s10–s12), (2) full assembled DOCs (Figures 3D–G and s13–s20), and (3) DOCs without the spring, in which the lids are connected only along one edge of the capsule (Figure 3H).

**Figure 3:**
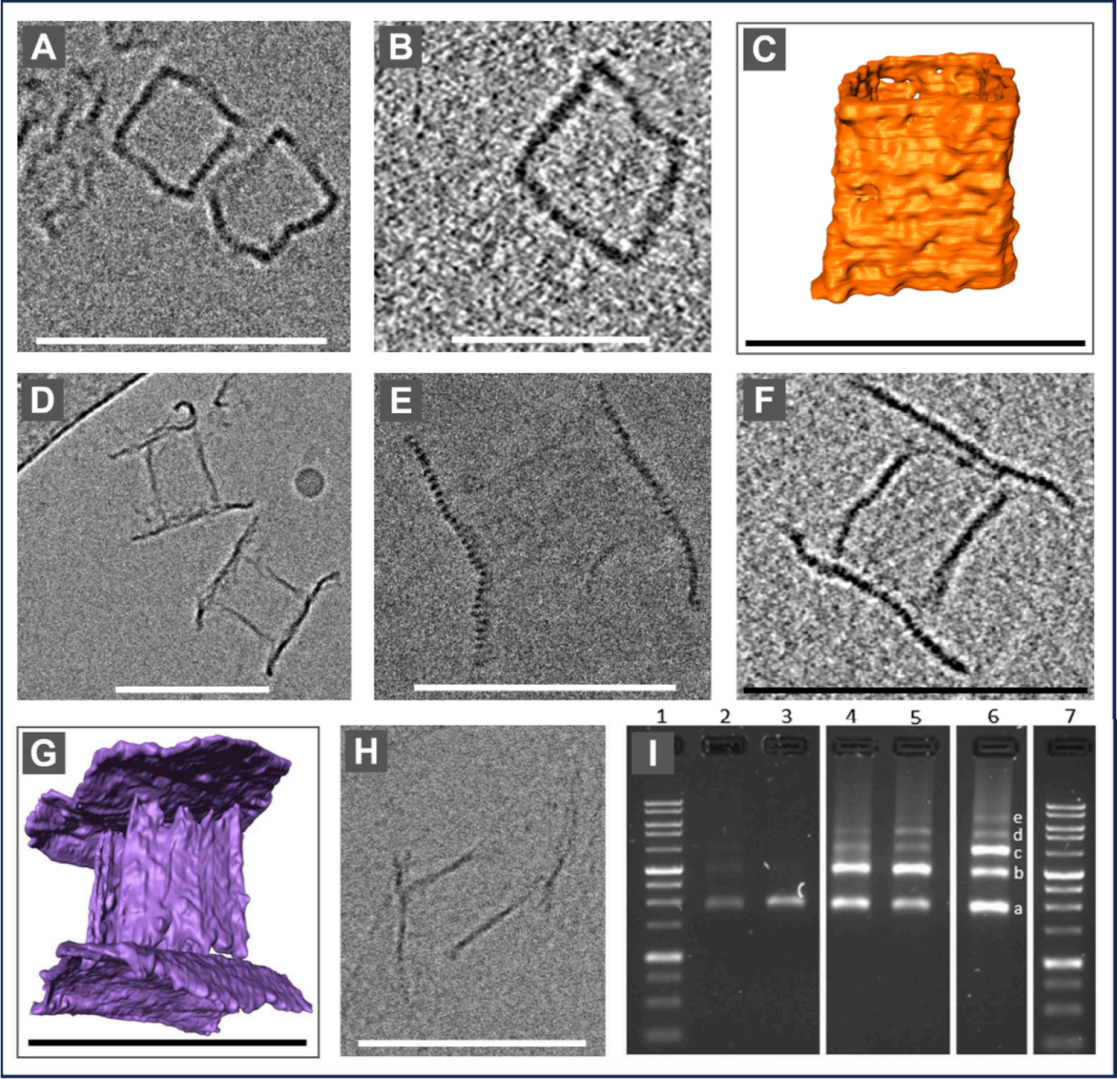
Cryo-TEM results of the DOCs. Scale bars are 100 nm for parts A and C–H and 50 nm for part B. (A) Cryo-TEM image of the boxes without lids. (B) Slice from a tomogram reconstruction of a capsule without lids. (C) Rendered segmentation of the capsule without lids from the tomogram in part B. (D and E) Cryo-TEM image of fully assembled DOCs (boxes with lids). Part E clearly shows the edges of the parallel DNA helixes of the lid. (F) Slice from the tomogram reconstruction of the full structure. (G) Cryo-TEM tomography of the capsule. Two of the box’s faces cannot be seen due to the missing wedge effect (see Methods and Figures s16, s18, and s20 for reconstructed images. (H) Image of an open capsule where the lids are connected only from one edge and there is no spring. (I) Gel electrophoresis. (1) 7:1 kb ladder, (2) box only, (3) lid only, (4 and 5) capsule with one lid, (6) capsule with both lids; (a) free boxes and lids, (b) capsule with one lid only, (c) full DOC, and (d and e) aggregates.

The DOC’s dimensions as measured by AFM and cryo-TEM closely match the caDNAno design. AFM measurements showed lid dimensions of 90 ± 4 nm × 73 ± 3 nm (Figure s21) and capsule dimensions of 65 ± 4 nm × 53 ± 1 nm wide (Figure s2). From cryo-TEM tomograms, the lid dimensions are 91 ± 8 nm × 62 ± 8 nm, while the capsule height is 62 ± 2 nm and its width is 36 ± 1 nm (Figure s23). The fully assembled structures (Figure 3D–G) also agree well with the design, appearing as a hollow, square-like box sealed on both sides by rectangular lids, confirming the effectiveness of the hinges and springs in connecting the lids to the capsules and maintaining closure. Gel electrophoresis (Figures 3I, s24, and s25) further confirmed high assembly yield and uniformity.^25^

The ability of the springs to keep the capsules securely closed under normal blood flow yet open in response to elevated shear forces within narrowed vessels, is critical for the intended drug delivery application. Opening a hinged lid in a low-Reynolds-number flow is analogous to classical analyses of rotating plates or hinged flaps in viscous flow (Figure M7), where the hydrodynamic load arises from the combined action of normal pressure and tangential viscous stresses.^26,27^ An analytical solution for this problem exists only for idealized geometries.

For thin structures the hydrodynamic torque is dominated by the pressure (normal) component of the load rather than by tangential shear. Therefore, for simplicity, we approximate the distributed hydrodynamic load as an effective force *F*_eff_ acting at a distance of order *a* (the lid size) from the hinge. Since the spring attaches at a comparable distance from the hinge, opening occurs when *F*_eff_ exceeds the elastic restoring force supplied by the spring. In a low-Reynolds-number shear field, the hydrodynamic force on a small object near a wall is given by *F* = *C*μ*Ga* ^2^, where μ is the viscosity, *G* is the shear rate,^28^ *C* is a geometric factor that depends on the distance from the wall and orientation in the flow, and *a* is the characteristic dimension of the object exposed to the flow. Using typical values for blood viscosity and a capsule with *a* = 50 nm located about *d* = 75 nm from the wall, the estimated force is approximately 3–4 pN at a wall shear rate of 10^4^ s^–1^, which is representative of severe stenosis.

Elongational flow at the entrance to a stenosis can further increase the tensile load.^29,30^ Under normal arterial conditions, where wall shear rates are closer to 250 s^–1^, the force is roughly 40 times smaller, on the order of 0.1 pN. Although exact values depend on local geometry and flow conditions, these estimates provide a physically grounded reference for the spring design,^31^ showing that the spring must remain closed under subpiconewton forces while opening at a few piconewtons.

Achieving the required mechanical response at the ∼50 nm scale is not feasible with dsDNA, whose persistence length (∼50 nm) is comparable to the capsule dimension, thus it acts almost as a stiff rod, leaving little room for further extension under the low forces generated by physiological and pathological shear. We therefore chose to use ssDNA which has a persistence length of ∼1 nm and remains compact at rest yet extends substantially under forces of 3–5 pN. In addition, ssDNA forms stem-loop structures (Figure 4B) that shorten its effective contour length and introduce intrinsic tension^32^ that helps to keep the lids sealed under normal flow.

**Figure 4:**
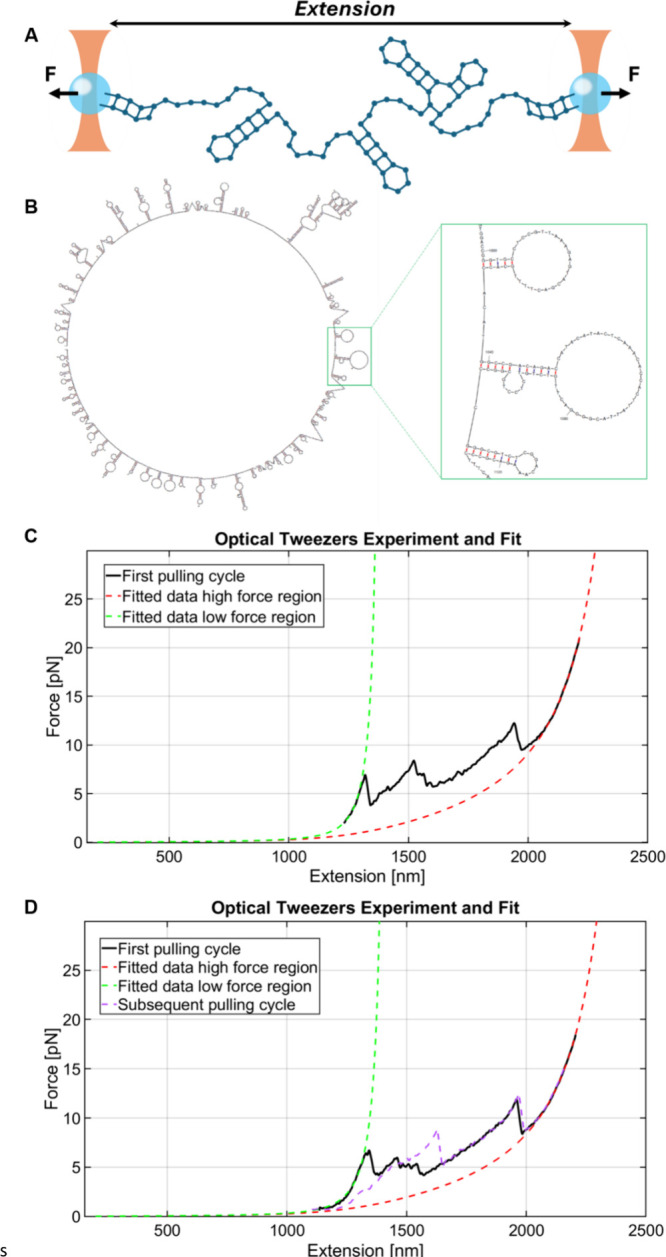
Characterization of the spring’s mechanical properties using optical tweezers. (A) Schematic representation of the stretching force–extension experiments. (B) Possible conformation of the ssDNA with a set of stem loops as predicted by mfold, under the conditions of 25 °C, 1.5 mM Mg^2+^, and 150 mM Na^+^. For more optional configurations, see Figure s26. (C and D) Optical tweezers force–extension curves. The first stretching cycle is shown in black, and a subsequent one in purple. Red and green dashed curves show WLC curves fitted to the high and low force regions, corresponding to the full construct without stem–loop structures (red) and with them (green), respectively. The dwell time between each pulling cycle is 30 s.

This property is also advantageous for the assembly of the structure; when the box, spring and lids are incubated together under a mild thermal gradient, the stem loops are open, and the increased length allows efficient hybridization. Then, loops gradually reform upon cooling, so that the effective length and stiffness of the spring are resumed.

On the basis of the required elastic properties of the spring, we chose a 2049 nt ssDNA segment amplified from lambda DNA. The first 25 nt at each end form dsDNA for anchoring, leaving 1999 nts ssDNA (Figure 4A). The full contour length of this ssDNA is approximately 1200 nm, assuming 0.6 nm per nucleotide.^33^ To understand its natural conformation under zero or very low force, we first consider the root-mean-square end-to-end distance of an unstructured ssDNA chain, $\sqrt{\left\langle R^{2}\right\rangle }\approx \sqrt{2L_{0}L_{p}}$ , where *L*_0_ is the nominal contour length and *L*_p_ ≈ 1 nm its persistence length, which gives approximately 49 nm. This is comparable to the dimensions of the capsule. In practice, the effective contour length is shorter because of stem-loop formation. mfold predictions^34^ (Figure 4B) yield an ensemble of possible folds and indicate that in the lowest free-energy structures only 158 ± 54 nt remain unpaired. However, not all stem loops are expected to be stable under the conditions relevant to our device,^35,36^ but the net effect is that the effective end-to-end distance is shorter than the 47 nm separation between the anchoring points, ensuring that the spring is under pretension and that the lids remain sealed under normal flow.

Under pathological flow, the shear-induced force acting on the lid is expected to stretch the spring. The elastic response of the ssDNA can be described by the wormlike chain (WLC) model^33^

$$\frac{FL_{p}}{k_{B}T}=\frac{1}{4}{\left(1-\frac{x}{L_{0}}\right)}^{-2}-\frac{1}{4}+\frac{x}{L_{0}}$$

where *F* is the force, *k*_B_ the Boltzmann constant, *T* the temperature, and *x* the extension. A more accurate model is the extended wormlike chain (eWLC) model^37,38^ that accounts for the intrinsic elasticity of the polymer. Nevertheless, in the low-force regime relevant to our system, the use of the eWLC model leads to a negligible difference, see Supplement section M10. These considerations suggest that forces in the range of a few piconewtons should be sufficient to extend the spring beyond the 47 nm lid–box spacing and thereby open the capsule, a prediction that we evaluate quantitatively in the next section.

To directly assess the spring’s force–extension properties, we performed single-molecule measurements using a high-resolution dual-trap optical tweezers setup (Figure 4A).^39,40^ The measured construct consisted of the 2049 nt ssDNA spring flanked by two 2000 bp dsDNA handles, each modified for specific attachment to antidigoxigenin or streptavidin-coated microspheres that are trapped by the optical tweezers. A typical force–extension curve (Figure 4C,D, black trace) begins with a gradual rise in force and extension, followed by abrupt force drops accompanied by increases in extension. These steps correspond to sequential unfolding of stem–loops, whose position and size vary between molecules (Figures 4C,D and S27–S30). After the ssDNA is fully unfolded, the force increases smoothly above ∼15 pN, consistent with the elastic response of a fully extended ssDNA segment flanked by dsDNA handles. Subsequent stretching cycles differ from the initial one (Figure 4D), reflecting the formation of new stem–loop structures during relaxation (Figure 4D, black vs purple, and Figures S28–S30).

The high-force region was fitted using three serially connected polymers (the fully unfolded ssDNA segment and the two dsDNA handles), each described by the WLC model (eq 1). The fitting procedure included two free parameters: an extension offset and the ssDNA persistence length, Lp_ss_. All other parameters were fixed and includes a persistence length of Lp_ds_ = 50 nm^42^ for the dsDNA, the contour lengths of dsDNA taking a base-pair separation of 0.34 nm/bp and the ssDNA contour length taking a nucleotide width of 0.6 nm/nt.^43^ Example fits are shown in Figure 4C,D (red dashed line). The distribution of extracted Lp_ss_ values is presented in Figure 5A (left panel), with a mean value ${\mathrm{Lp}}_{\mathrm{ss}}=0.79\pm 0.08$ nm, in good agreement with previous reports.^38^ Using these Lp_ss_ values, we fitted the preunfolding regime to obtain the effective ssDNA contour length *L̅*_0_ which varies between molecules due to differences in stem-loop configurations. Representative fits are shown as dashed green lines in Figure 4C,D, and the resulting distribution is shown as boxplot (Figure 5A). Notably, these *L̅*_0_ values correspond to *n* = 328 ± 161 nts, consistent with the mfold predictions and previous work.^35^ Traces lacking distinct steps likely reflect gradual unfolding of small loops and were excluded.

**Figure 5:**
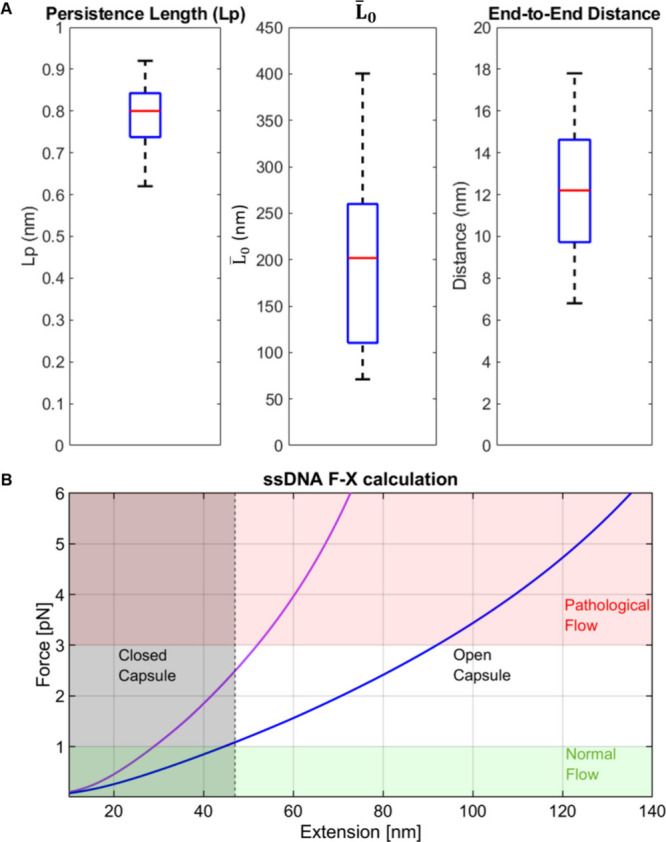
Analysis of the force–extension results. (A) Boxplots of Lp_ss_, *L̅*_0_, and the end-to-end distance (related to*L̅*_0_) of the ssDNA springs. These distributions result from the different stem loops that can be formed. The averages are 0.78, 197, and 12 nm, respectively. (B) Force–extension curves showing the 70% confidence interval limits of the measured springs for the ssDNA length *L̅*_0_ of 120 nm (purple) and 250 nm (blue). Forces below 1 pN correspond to healthy vessels (green background), whereas forces above 3 pN indicate pathological flow (red background). The gray dashed line marks an extension of 47 nm, the threshold at which the capsule opens.

Based on these results, we can evaluate the expected performance of the spring in the DOC (Figure 5B). To address the measured variability in *L̅*_0_, we used eq 1 to calculate force–extension curves for two extreme cases *L̅*_0_= 120 and 250 nm (Figure 5B, red, blue), spanning ∼70% of the measured springs (one standard deviation). At force values below 1 pN, which corresponds to up to 10 times the physiologically normal flow as calculated above, both curves show an extension below 47 nm (the capsule size), ensuring that the capsule remains closed. Given that the spring is partially stretched and constrained to an extension 47 nm in the closed DOC, this means that the spring will exert a tension of 1–2.5 pN opposing opening of the DOC by normal flow fluctuations. At forces exceeding 3 pN, which is typical of pathological flow in stenotic sites, the elastic response of ssDNA predicts an extension of 50–90 nm, indicating that the capsule will fully open. Notably, the length of ssDNA and the flanking dsDNA can be further adjusted to fine-tune the capsule’s performance under real physiological conditions.

In summary, we developed a mechanoresponsive DNA origami capsule that exploits the elastic properties of DNA to achieve shear-triggered drug release at sites of vascular narrowing. The device consists of a hollow capsule sealed by two large lids held together by a DNA-based spring which keeps the capsule closed under normal blood flow but stretches to open the capsule in response to the elevated shear forces in narrowed blood vessels.

These features of the system enable a targeted delivery strategy for potent therapeutics such as tPA while minimizing risks to healthy tissue. The capsule architecture was validated by AFM and cryo-TEM measurements, showing excellent agreement with the design. Considerable effort was invested in optimizing the properties of the DNA spring to achieve the required shear-response behavior. We used ssDNA for the spring, as a dsDNA cannot provide the necessary elastic properties at the nanometer scale of the capsule.

We further validated the assembly and mechanical performance of the ssDNA spring using optical tweezers, confirming both its robustness and the required force–response behavior. The design is highly modular, allowing precise tuning of spring stiffness, lid geometry, and capsule volume, as well as integration of targeting ligands, protective coatings, or a wide range of therapeutic payloads.

Although our primary focus is thrombosis, this strategy is broadly applicable to other conditions involving altered hemodynamics, including vasospasm and additional cardiovascular pathologies. Ongoing efforts aim to load and stabilize therapeutic formulations within the capsule and to evaluate drug release under physiologically relevant flow conditions, including biomimetic microfluidic systems that recapitulate vessel geometry and near-wall shear environments.

## Supplementary Material



















## Notes

Details of the DNA origami structures design and preparation, TEM, AFM, and agarose gel images, and additional optical tweezers experiments (PDF)
Video movie of the DNA origami structure and tomogram (MP4)
Video movie of the DNA origami structure and tomogram (MP4)
Video movie of the DNA origami structure and tomogram (AVI)
Video movie of the DNA origami structure and tomogram (AVI)
Video movie of the DNA origami structure and tomogram (AVI)
Video movie of the DNA origami structure and tomogram (MP4)
Video movie of the DNA origami structure and tomogram (AVI)
Video movie of the DNA origami structure and tomogram (MP4)
