# Supplementary material for: Engineering a Mechanoresponsive DNA Origami Capsule for Drug Delivery to Narrowed Arteries
Source: Nano Lett. 2026 Jan 5;26(6):1988–94. doi: 10.1021/acs.nanolett.5c04066 (PMC12922184; doi:10.1021/acs.nanolett.5c04066)
Supplement: Supplementary file 1 [file nl5c04066_si_001.pdf]

Supplementary Information for

Engineering a mechano-responsive DNA origami capsule for drug delivery to narrowed arteries

Hadas Omer[1,2], Hadeel Khamis[3,4], Zipora Lansky[5], Racheli Boeangiu[1], Netanel Korin[1], Ariel Kaplan[1,2,3, *] and Yuval Garini[1,2, *]

[1] Faculty of Biomedical Engineering, Technion – Israel Institute of Technology, Haifa, Israel

[2] Russell Berrie Nanotechnology Institute, Technion − Israel Institute of Technology, Haifa, Israel

[3] Faculty of Biology, Technion – Israel Institute of Technology, Haifa, Israel

[4] Faculty of Physics, Technion – Israel Institute of Technology, Haifa, Israel

[5] Department of Chemical Engineering, Technion – Israel Institute of Technology, Haifa, Israel

[*] Corresponding authors

Supporting Materials (Supporting M. 1 to 8)

M1. DNA Origami Design
M2. DNA Origami Assembly
M3. DNA Agarose Gel Electrophoresis
M4. Atomic Force Microscopy (AFM)
M5. Cryo-TEM
M6. Spring preparation
M7. Spring's design
M8. Mfold34 models
M9. Optical tweezers measurements
M10. WLC vs extended WLC

Supporting Figures (Supporting Fig. 1 to 29)

S1. caDNAno design - lid
S2. caDNAno design - box
S3. Explanation of the caDNAno lid's designs
S4. Explanation of the caDNAno box's designs
S5. Lids - AFM
S7. Open capsule - AFM
S8. DOCS – AFM
S9. SNUPI model of the DOC - video
S10. Boxes Cryo-TEM image
S11. Box Cryo-TEM image
S12. Box Cryo-TEM tomogram video
S13. DOC along z axis under the cryo-TEM video
S14. DOC together with free boxes and lids along z axis under the cryo-TEM video
S15. DOC together with free boxes and lids along z axis under the cryo-TEM video
S16. DOC Cryo-TEM tomogram (with hand corrections) - video
S17. DOC cryo-TEM Tomograms
S18. DOC cryo-TEM Tomograms (with hand correction)
S19. DOC along z axis under the cryo-TEM video
S20. DOC's Cryo-TEM tomogram video (with partial hand corrections)
S21. Lid dimensions measurements AFM
S22. Box dimensions measurements AFM

S23. DOC dimensions measurements Cryo-TEM
S24. DOCH Gel electrophoresis
S25. DOCS Gel electrophoresis
S26. MFOLD simulations
S27. Force-extension curves of one spring
S28. Force-extension curves of one spring
S29. Force-extension curves of one spring

Supporting Tables (Supporting Table 1 to 14)

T1. Native DNA staple strands of the box
T2. Box's staples for hinges conjugations with the "up" lid
T3. Box's staples for hinges conjugations with the "down" lid
T4. Box's staples for no hinge's conjugations with the "up" lid
T5. Box's staples for no hinge's conjugations with the "down" lid
T6. Box's staples for spring conjugations
T7. Native staple strands of the lids
T8. Additional native staple strands of the "up" lid
T9. Additional native staple strands of the "down" lid
T10. "Up" lid's staples for hinges conjugations
T11. "Down" lid's staples for hinges conjugations
T12. "Up" lid's staples for no hinge's conjugations
T13. "Down" lid's staples for no hinge's conjugations
T14. Staples for spring conjugations – both lids

Supplementary Material

Methods

1. **DNA Origami Design**

caDNAno[1] was used to design the DNA origami box capsule and lids separately, by square lattice. Staple sets are available in supporting information. SNUPI[2] software was used for creating predicted models of the structures before the assembly. In order to prevent structures aggregation, we used t-tails that prevent stacking interactions[3]. In DNA origami design, curvature often arises from intrinsic mechanical stress within the structure. This can be mitigated by selectively shortening a subset of staples at specific sites to relieve tension and improve planarity (Figure s1-s3). To mitigate inner stresses and ensure a flatter structure, deletions were introduced in the caDNAno design. Deletions remove bases from specific staple strands, locally adjusting the helical periodicity and relieving internal tension. This helps to counteract unwanted curvatures, leading to a more planar DNA origami structure. The structures were subsequently refined through several iterations to achieve sufficiently flat lids, thereby ensuring full closure of the capsules[4]. If necessary, larger structures can be synthesized to increase payload capacity.

2. **DNA Origami Assembly**

The lid: We used p7249 plasmid (Tilibit) as scaffold. The assembly reaction was performed in TAEx1 buffer (Bio Lab) with 12mM MgCl2 (Sigma). 20nM p7249 and ten- or five- fold excess of each staple (IDT) (only specific staples that have some function as binding to something were in fivefold excess). The sample was heated to 65°C for 15min and cooled from 60°C to 44°C in steps of 1°C every 15min in PCR (Bioer). The box: We used p7560 (Tilibit) plasmid as scaffold. The assembly reaction was performed in TAEx1 buffer with 12mM MgCl2. 20nM p7560 and ten- or five- fold excess of each oligonucleotide (only specific staples that have some function as binding to something were in fivefold excess). The sample was heated to 70°C for 15 min and cooled from 65°C to 25°C in steps of 1°C every 15 min in PCR. Free boxes and lids were purified using AMICON 100K kit after assembled. Binding reaction between the capsule and the lids: We assembled the box and the two lids separately, in 3 separated reactions,

and purified each of them from the excess staples using AMICON 100K kit (Merck) with TAEx1 buffer containing 100 mM NaCl and 6 mM MgCl2. The binding reaction between them differentiates when we create the DOCH or the DOCS. The DOCH: We mixed the box with the lids in 1:1:1 concentration ratio, when each of them is in the concentration of 10 nM in the final reaction of 50 µl. For the binding, the sample was heated to 30°C for 2 h and cooled from 29°C to 25°C in steps of 1°C every 1 min in PCR. The DOCS: We mixed the boxes with the lids in 1:1:1 concentration ratio and added a "blocker" staple to bind partially to the box's steaky ends for the spring's binding, in PCR reaction (35°C for 10 min and cooled from 34°C to 31°C in steps of 1°C every 1 min, 30°C for 2 h and cool from 29°C to 25°C in steps of 1°C every 1 min). Then, we added the springs to bind only through the lid's steaky ends using a thermal gradient (35°C for 2 h, then cool from 34°C to 25°C in steps of 1°C every 1 min). Then, we added "anti-blocker" staples to release the blockage boxes, allowing the spring to connect at both ends in PCR reaction (37°C for 2 h, then cool from 36°C to 25°C in steps of 1°C every 1 min). reaction's concentrations: the box and lids were mixed to form 50 µl solution with 10 nM of each and 1 µl of the "blocker" staple of 5 µM was added. Springs were added so their reaction concentration was ten-fold higher than the box's. "Anti-blocker" was added so its reaction concentration was five-fold higher than the "blocker."

3. **DNA Agarose Gel Electrophoresis**

The assembled structures were analyzed using 1% agarose (Serva) gel electrophoresis (Cleaver Scientific - UK) in 1X TAE buffer with 6mM MgCl2. The samples (10µl) were mixed with loading dye (x6) (2µl, GoldBio) and loaded into the wells. The gels were run at 70 V for 1.5 h in ice. A 1 kb DNA Ladder (GoldBio) was used as a reference standard. DNA bands were visualized by adding SYBER-SAFE (ThermoFisher) to the gel solution for staining. Each sub-structure (box and lids) in the assembly reaction for the complete capsule (DOCH/DOCS/open capsule) was used at a concentration of 10 nM (open capsule: Figure 3I - main manuscript; DOCH and DOCS: Figures S24 and S25). Even the hinges in the opened capsule show strong binding efficiency, indicating effective hinge assembly. Their band (Figure 3I - main manuscript, lane 6 - band c) is both intense and sharp, indicating high binding efficiency, strong assembly quality, and uniform structures.

4. **Atomic Force Microscopy (AFM)**

Sample preparation: We deposited 10ul of the samples in a concentration of 0.5-0.8nM on a freshly cleaved mica surface (Getter group) and left to adsorb for 5 min. The sample was diluted to this concentration with TAEx1 buffer containing 14mM MgCl2. 150 ul TAEx1 buffer containing 14mM MgCl2 was added above the mica for liquid imaging. Imaging: Images were acquired at room temperature using Bruker NanoWizard ULTRA Speed AFM operated in PeakForce Tapping mode using SNL-10-A, and FASTSCAN-D cantilevers (Bruker).

5. **cryo-TEM**

Sample preparation for Cryo-TEM tomography: Grids for sample support were either 200 mesh copper overlain with lacey carbon film, or Quantifoil 200 mesh copper grids with 2/2 holes. Grids were glow discharged for 1 minute at 15 mA prior to sample application. Fiducial gold beads (10 nm) were added to the sample at a dilution of 1µl fiducials to 20 µl sample. For sample vitrification a grid was placed in the controlled environment chamber of the GP2 automatic plunge freezer (Leica), which was set to 22ºC and 90% humidity, a 3 µl drop of sample was applied to the carbon side of the grid and ½ ul of sample was applied to the copper side of the grid, then the grid was blotted for 5 seconds from the copper side before being plunged into the liquid ethane. Vitrified samples were stored in liquid nitrogen until imaging.

Cryo-TEM imaging and tomography: Cryo-TEM images and tomograms were collected on Talos F200 microscope (Thermofisher Scientific) equipped with a Schottkey FEG and operated at 200 kV. Gatan 914 cryo-tomography holder was used to transfer the samples into the microscope under cryogenic conditions. The microscope was set to nanoprobe mode, spot size 5, with a 70-micron objective

aperture. 2D images were collected in low dose mode on Falcon III direct electron detector at around -1 µm defocus. Tomograms were collected using Thermofisher Scientific Tomography software, in a dose-symmetric collection tilt scheme starting from 0º tilt and increasing at $\pm 2^0$ increments until $\pm 70^0$. Tilt series images were recorded on a Falcon III direct electron detector (Thermofisher Scientific) at 57kx magnification, pixel size of 0.2 nm, and at -5 µm defocus. Total electron exposure dose per tomogram was 114 e/A2. 3 tomograms were collected from two different samples, with a total of 9 capsules.

Tomogram processing: Tomograms were reconstructed in the IMOD package, using gold fiducial beads for alignment, and Back Projection with SIRT-like filter equivalent to 50 iterations for computing the reconstructions. For visualization of tomogram z-slices, every 20 slices were averaged to increase the signal-to-noise ratio. Segmentation of capsules were done in Amira 3D 2024.1 (Thermofisher Scientific). Prior to segmentation tomograms were filtered (gaussian, recursive exponential, anisotropic diffusion, and FFT bandpass filtering). The filtered data was used to create an initial rough segmentation using intensity threshold. To create a refined segmentation, the rough segmentation was selected at every 5 slices over the raw data or over very lightly filtered data, and manually corrected by filling holes (using the raw data as reference), while the slices in between were filled by interpolation. The sides of the capsule parallel to the grid are obscured due to the missing wedge artifact. Rendering and animation of segmented data was done in Amira software.

6. **Spring preparation**

ssDNA was generated as previously described43. A 2049 bp dsDNA segment was amplified via PCR (Bioer) using lambda DNA (New England Biolabs) as a template, and purified using a PCR purification kit (QIAGENE). The dsDNA was then digested Lambda Exonuclease (Biolabs) to generate ssDNA, which was purified using a MINELUTE PCR purification kit (QIAGENE).

The full spring's sequence:

5' –
TCAGCTTGCCCCTCAGCGATGACCTCAGCAAGGACCAGCGTTTTGTTGAAATCGGACAGGGTTGAG
TTGCCCTGATACCAGGCATACGCCAGCGCACCGGTCGCCACCGCCAGCGAGGTGGCCCCCACCATC
GGCAGGGTGATCGCACCGGCAAGCCCCCTGAACATGGGGATCATCCCGCCGAAGGAGTCCTTCACC
TGCCCCCCCTGTTGCAGCAGGATCAGCCACGGACTTTGCCCGCCTGCAAGCTGCGTGGCCACGTCG
GTGAACTGTGCAGGCAGCATACGCATGGCGGCTTTATACTGCCCGACGGAAATCCCCGCTTTCTGT
GCAGCCAGCGCCTGTCGGCTCAGCGACTGTTCAACGACTGCCGCTGTTTTTTTCGCATCACTTTCC
GTACCAGAAAAATGACGCCTGACTCTGGCCATCTGCTCGTCAAATCTGGCCGCATCCAGACTCAAA
TCAACGACCAGATCGCCTACCGGTTCAGCCATACCGGACTCCTCCTGCGATCCCTTCTGATACTGT
CATCAGCATTACGTCATCCTCCGTCATGTCCGCCACATCCGGGGAAGCGGGGATAACTTCATTCCC
GTCCGGGCCAAAGCGGACACCTCCGGCAAGCCCTGCCGCTTTCTGCATCAGCACATCATCTTCAGG
CTCTTCGTCAGCCTCGCGCCGGTTCAGCAGACTGAAATCCAGCGGATGCATATCCGGATCGCTGAA
AAACAGGCTGAGCACGGTGTACGTCAGCCCGGAAAAGTGCATATCCAGCAGAACATCATGAAAATA
ATGGGTACTGTAAAAGCGGTGCCAGTCGGCATACTCCGTGGATGACATCCCGGCAAGCATGGCACG
CCAGTCGGGTCGCCCCATCTCACGCGCCAGTTTCAGGGCAAAACTCAGCTCACCGTCGAACACTTT
CCCGCAGAAACAGGCTCTGCGGGCCCGGCGTCCTCTGTCTGTTCAGGGGCATTATTCACCACAAAC
TCATACATACCAGACAGCCGGTACACCACGTTTTCAGCATGAGAAATTGCCTCCGTGGGCCAGGTG
GTAAGCACTTCCTGCTCAATCTGTTTAACGGCTTCATTCATGGACGGCATCTGCGTCTTCTGCGGA
TGGTTATGCCACAGGGACATCGCCACCAGAAACGCGCCGGTTCTGATGGCGTCTTCCACAGTAAAC
TTCCGGTTGCTGTCTGACTCCGCCTGTTCTGCCTGCCGTTTCATCAGGGCGAGATGCTCAATGCGC
TGCAGGGCTGACAGTTCAGAAAGCGTGACGGTCACACCGTTATGTTCAAATGATTCGGTTTTCAGG
AACATCGCTGACTCTCCGGATTAACTGGCGGTGACGGTAATTTCTGCAACCGCAGCAAACTCACCA
TTACCGGATACAACCGGAATGTTGACCTTGCCTGCAGCAACGCCGTTCACGGTGATGGTCATACCA
CTGACCGACACGGTGGCTTTTGTTTTATCCGCAGACACCGCACGAAAGCTCTTGTCGGTTACGCCC
TCCGGCTGGAAGGCCACGGTCAGCGTGGTGCTCTGCCCTTTCACCACCGAGGTGCTGGCAGGCGTC
ACGGTCATGCCGGTTGCCGCTGTTACCGTGCTGCGATCTTCTGCCATCGACGGACGTCCCACATTG
GTGACTTTCACCGTGCGGGTGATCACTTCCTTCGCCGTCACCGCCTTACCGATACTGCTGACCCAG
CCACGGAACACATCGACCGTGCCGTTCGGGAAGCGGATTTTATAGGCACGGGTATCGCCTTCATTA
AACCACGCCAGCAGCGCCTGCTGCCCCTGCTCTCCGGGCATCCACGCCAGCGTGAAGCTGGTATCT

CCGGCAGATTTCTGCCCCTGCCCGGTCGCAGTCCAGTCTGCATCTTCATCATCGAGATAGCTGTCG
TCATAGGACTCAGCGGTCAGTTCGCCGGGCGTCAGGTCTTTAACTTTTGCCAGACGCGACCAGTCA
ACGTCTGAAAGCGGATTCGCGTAAGGGTCACCGCTCCCCTTATAAACCCAGGCTCGCCGTCTAGGC
CAT
-3’

7. **Spring's design**

The process of opening a hinged lid in a Stokes flow involves torque generated by tangential hydrodynamic stresses. The moment balance (Figure M7) provides a simple design presents that main forces and torques that act on the capsule lid. The full “lid-opening” problem is analogous to classical analyses of rotating plates or hinged flaps in viscous fluids, which treat the hydrodynamic load as a viscous torque arising from tangential shear stress but for which analytical solutions exist only under idealized geometries (e.g., O’Neill & Stewartson, J. Fluid Mech. **27**, 1967; Katz et al., J. Fluid Mech. **72**, 1975).

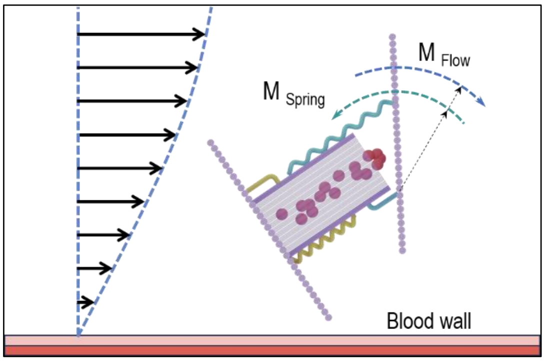

Figure M7| Schematic of the moment balance for an inclined, fixed system.

8. **Mfold34 models**

Simulations were performed under the conditions of 25°C, 1.5mM Mg+2, 150mM Na+1.

9. **Optical tweezers measurements**

Experiments were performed in a custom-made dual-trap optical tweezers apparatus, as previously reported37,37,44. The construct used in the optical tweezers experiment consists of a 2049-nt ssDNA strand flanked by two 2000-bp dsDNA handles, modified at one end with double digoxigenin or biotin, respectively. The dsDNA handles were generated using a standard PCR reaction (Q5® High-Fidelity DNA Polymerase) with lambda DNA (New England Biolabs) as a template and commercially synthesized 5’-modified primers. The handles were designed so that the non-tagged ends contain recognition sequences for the restriction enzymes NcoI-HF and BglI (New England Biolabs), which create 4-nt and 3-nt overhangs, respectively. The ssDNA, prepared as described earlier, was mixed in equimolar concentration with two short oligonucleotides designed to anneal to its ends leaving 4-nt and 3-nt overhangs complementary to those on the dsDNA handles. This mixture was phosphorylated using T4 Polynucleotide Kinase, then subjected to an annealing process in which the reaction was heated to 90°C and gradually cooled to 25°C over 3 hours. Finally, the full construct—including the two dsDNA handles, the ssDNA strand, and the annealed oligonucleotides—was assembled by ligation using a rapid ligase system (Promega) in a 1:1:1 molar ratio for 30 minutes at room temperature. To hold the construct between the traps, the complex was first incubated on ice for 15 minutes with 0.8 µm anti-DIG-coated polystyrene beads (Spherotech) and then diluted 1000-fold in working buffer (10 mM Tris-Cl pH 7.4, 150 mM NaCl, 1.5 mM $MgCl_2$, 3% v/v glycerol, 0.01% BSA). A tether was then formed in situ by trapping a DNA-bound anti-DIG bead in one optical trap and a 0.9 µm streptavidin-coated bead in the second trap, then bringing them together to allow biotin-streptavidin binding.

Notably, subsequent stretching curves differ from the initial one (Figure 4D, black vs. purple; Figures S28–S30), indicating the formation of different stem-loop structures during relaxation. After ~10 cycles, the structures no longer reform within the experimental timeframe (Figure S30), possibly due to trap-induced oxidative DNA damage[5].

10. **Comparison of WLC and Extended WLC Models**

    a. **Model description**

    The Extended Worm-Like Chain (eWLC) model is given by:

$$\frac{F \cdot L_p}{k_B T} = \frac{1}{4}\left(1 - \frac{x}{L_0} + \frac{F}{K_0}\right)^{-2} - \frac{1}{4} + \frac{x}{L_0} - \frac{F}{K_0}$$

    Where F is the force, x is the end-to-end distance, L_0 is the nominal length, L_p is the persistence length, $k_B T$ is the thermal energy, and K_0 elastic modulus of the polymer and accounts for changes in its intrinsic structure. In the extended WLC model, it accounts for the fact that the polymer backbone can be stretched beyond its contour length.

    b. **Typical parameters**

    For ssDNA, typical values are: $L_p = 0.79$ nm, $K_0 = 600 - 750$ nm (we used 675 nm), for our ssDNA spring L_0 varies between 120-250 nm.

    c. **Comparison with WLC**

    The figure below shows the standard WLC and the eWLC for the parameters above. At the low-force regime relevant to our experiments, the difference between the two models is negligible. Significant deviations appear only in higher forces, which are outside the physiological range considered here.

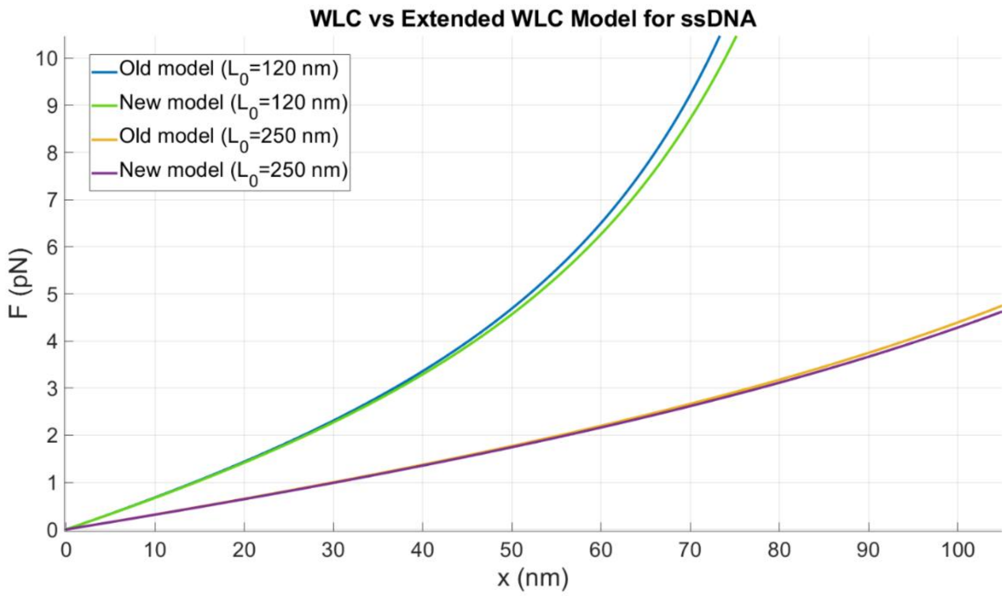

A.

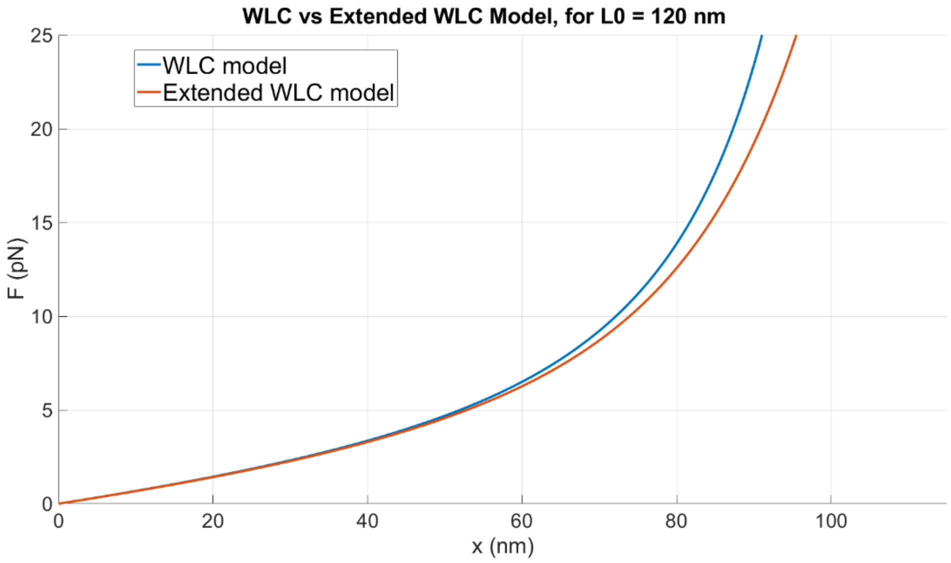

B.

Figure M1| Comparison of the standard Worm-Like Chain (WLC) model and the Extended WLC (eWLC) model. A. Comparison for two nominal lengths, L_0=120 nm and_L_0 = 250 nm. At the low-force regime relevant to our system, the difference between the WLC and eWLC is minimal, demonstrating that the simpler WLC approximation is sufficient. Significant deviations appear only at higher forces outside the physiologically relevant range. B. Comparison for nominal length of L_0=120 nm over a wider range of extensions. The difference between the models appears at larger forces.

Supplementary Figures

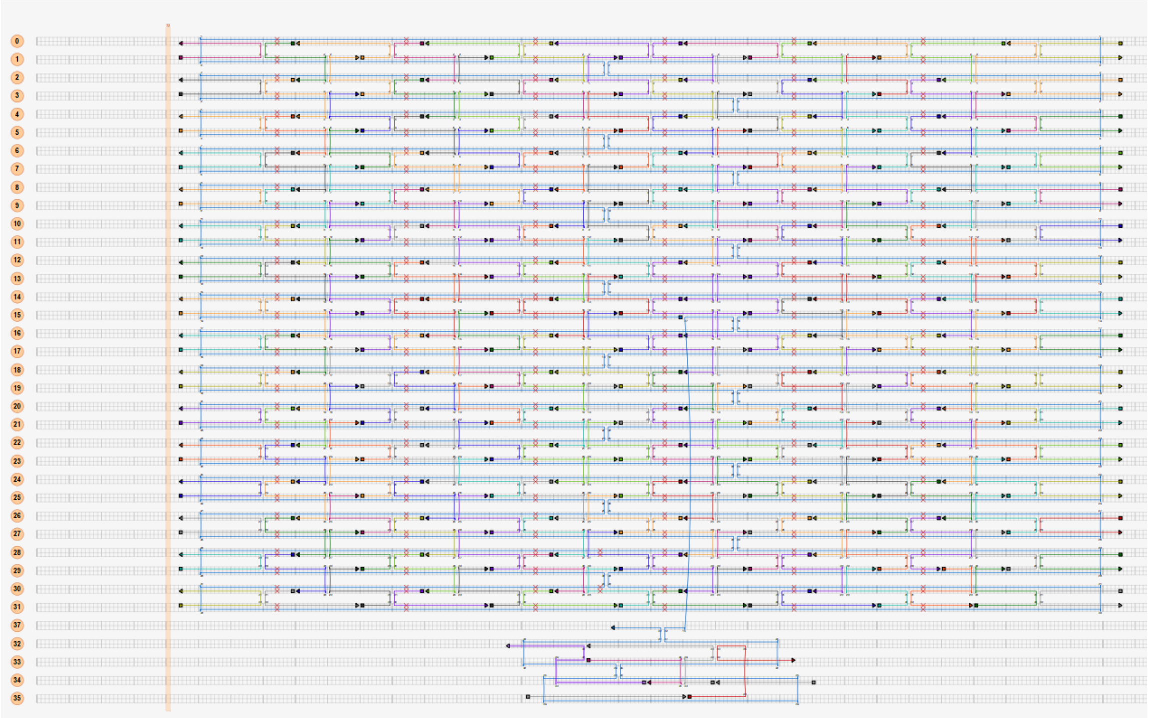

Figure s1 | The caDNAno design of the lid. The lid's length and width depend on one dsDNA helix's width (2.5-3 nm), and base-pairs gap. Its dimensions are 76 X 90 nm^2 and it is designed as a set of 32 dsDNA strands where each has 224 bp. Reducing the curvature is performed by shortening few staples at specific sites (deletions) – marked by red X. In DNA origami design, curvature can arise due to intrinsic mechanical stress within the structure. These stresses originate from slight mismatches between the periodicity of the staple strands and the natural helical repeat of DNA. DNA helices have a helical repeat of approximately 10.5 base pairs per turn, but when multiple helices are bundled together in an origami structure, maintaining perfect alignment across all helices becomes challenging. This cyclic mismatch leads to accumulated strain, causing unintentional bending or twisting.

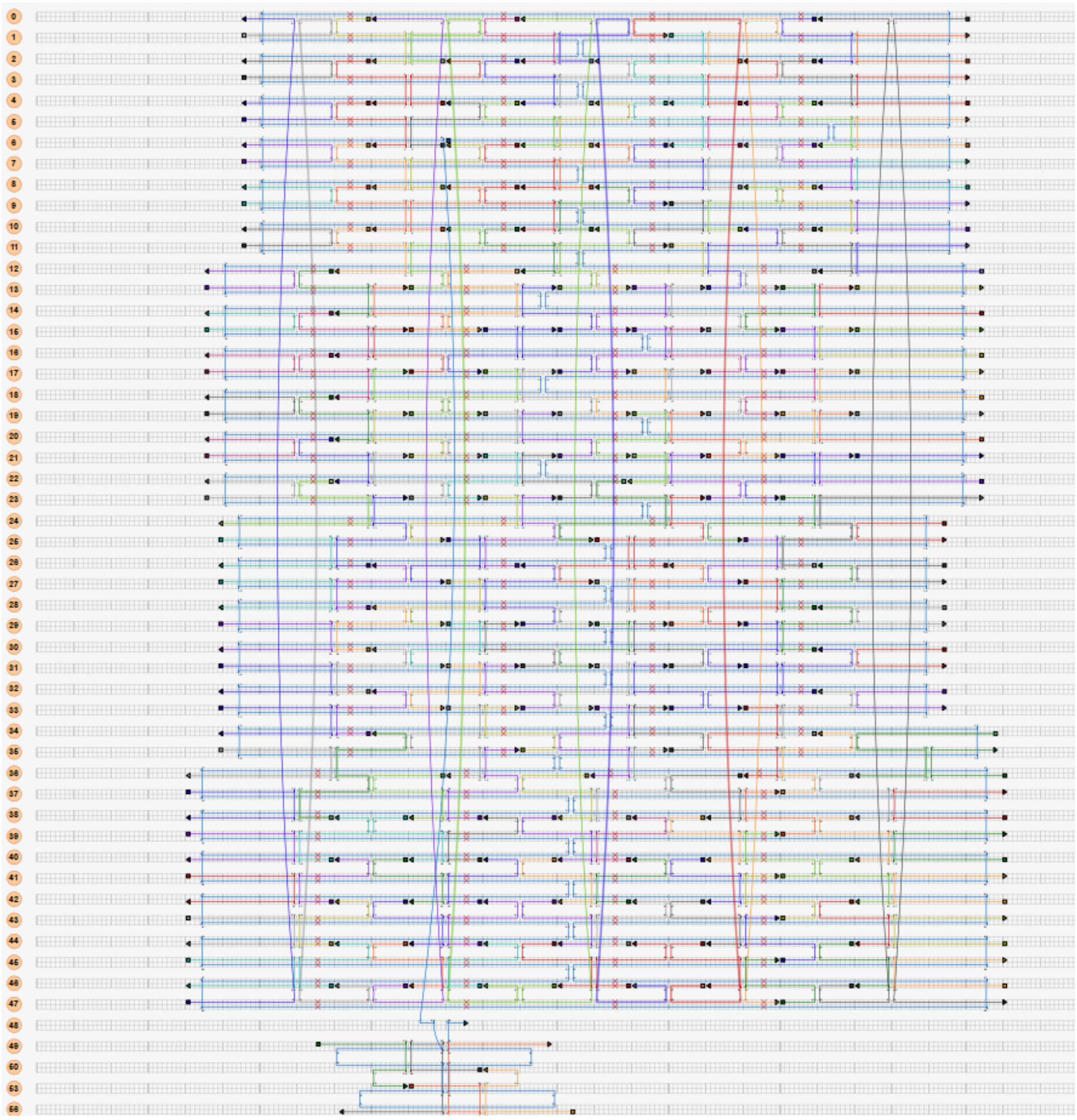

Figure s2 | The caDNAno design of the box. It is constructed of 47 dsDNA helixes where each has ~ 160 bp.

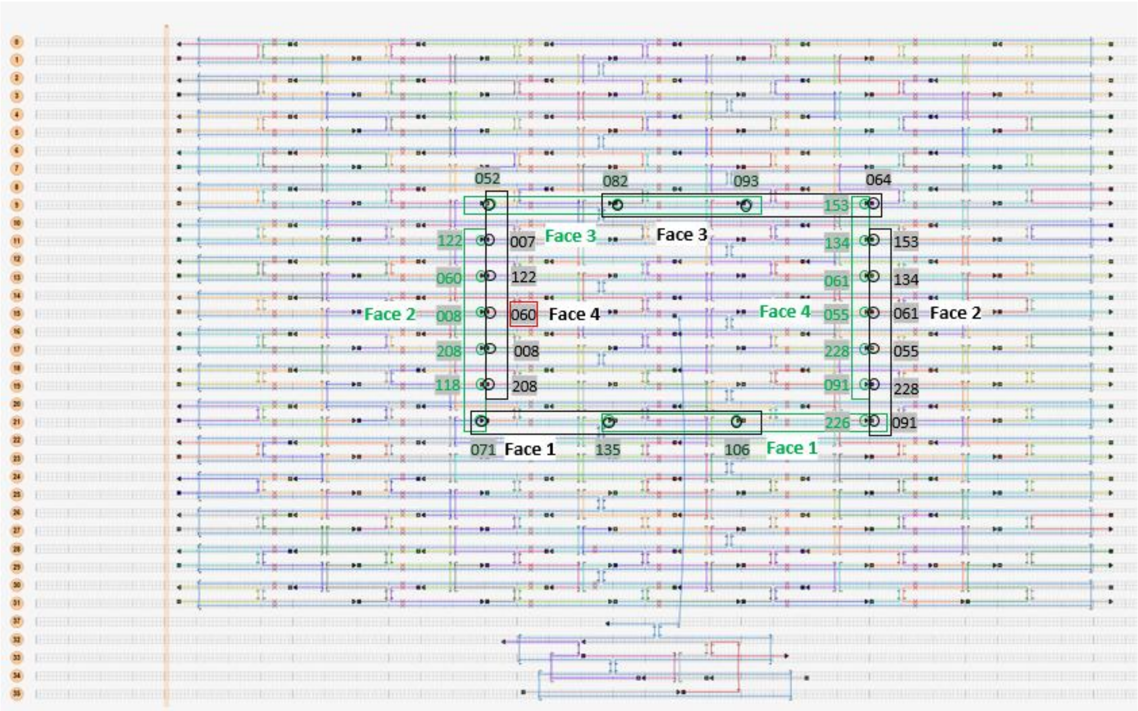

Figure s3 | Explanation of the lid's designs, by marking of the functional staples of the lids, upon the lids' caDNAno design. We will define the "up" lid and the "down" lid, which are differentiate of their special stales. ***Black*** *marks represent the "up" lid;* ***green*** *marks represent the "down" lid;* ***dark green*** *marks represent both lids. The rectangular frames (black and green) represent the face of the capsule when it is bonded to the lid (up and down respectively). The staples' edges Surrounded by a circle represent the exit of a steaky end from that edge of the stale, which create the connection to the capsule with complement steaky ends from the capsule (Figure s4 - main manuscript). The serial number of each staple is written aside with the matching color (see Table s10, Table s11). The faces of the capsule Figure s4 - main manuscript) are also mentioned along their rectangular mark). The red frame around "060" staple, represents that this is the edge of the steaky end for the spring connection for the DOCS (instead of the hinge steaky end).*

In the DOCH design there are a few hinges of each box face, when faces 2,4 contain 6 hinges and faces 1,3 contain 3 hinges (from each side of the capsule). Most of the hinges are hybridized in a zipper form, while 3 hinges (on face 1 for one edge and on face 3 for the other edge) are hybridized in a shearing form. Under increased shear-stress the hinges will dis-hybridized while opening the DOCH. Shearing hinges are stronger, allowing them to keep connected after the zipping ones disconnected. The DOCH were taken by Cryo-TEM microscope (**Error! Reference source not found.**(A)-(C) - main manuscript), a nd Cryo-TEM tomography were preformed (see supporting information, videos and Figures s20-s21). We also scanned the complete closed capsule complex via AFM (**Error! Reference source not f ound.**D,E) - main manuscript, and Figure s11).

In the DOCS Each lid is attached to the box capsule via stiff and short hinges through one face of the capsule (similarly to the DOCH), and via DNA-spring through the opposite face. In this structure, all hinges are in zipping form. The springs are forced to be 47nm long when it's trapped between the box capsule and the lid.

- *The missing staples in Figure 2.F in the main manuscript are: Lid_210, Lid_215, Lid_226, Lid_118 (downer hole); lid_152, lid_180 (upper hole).*
  Lid_226, Lid_118 are marked in Figure s3.
  Lid_210 – in gray, above 135.
  Lid_215 – in orange, above 106.
  Lid_152 – in gray, below 082.

Lid_180 – in pink, below 093.

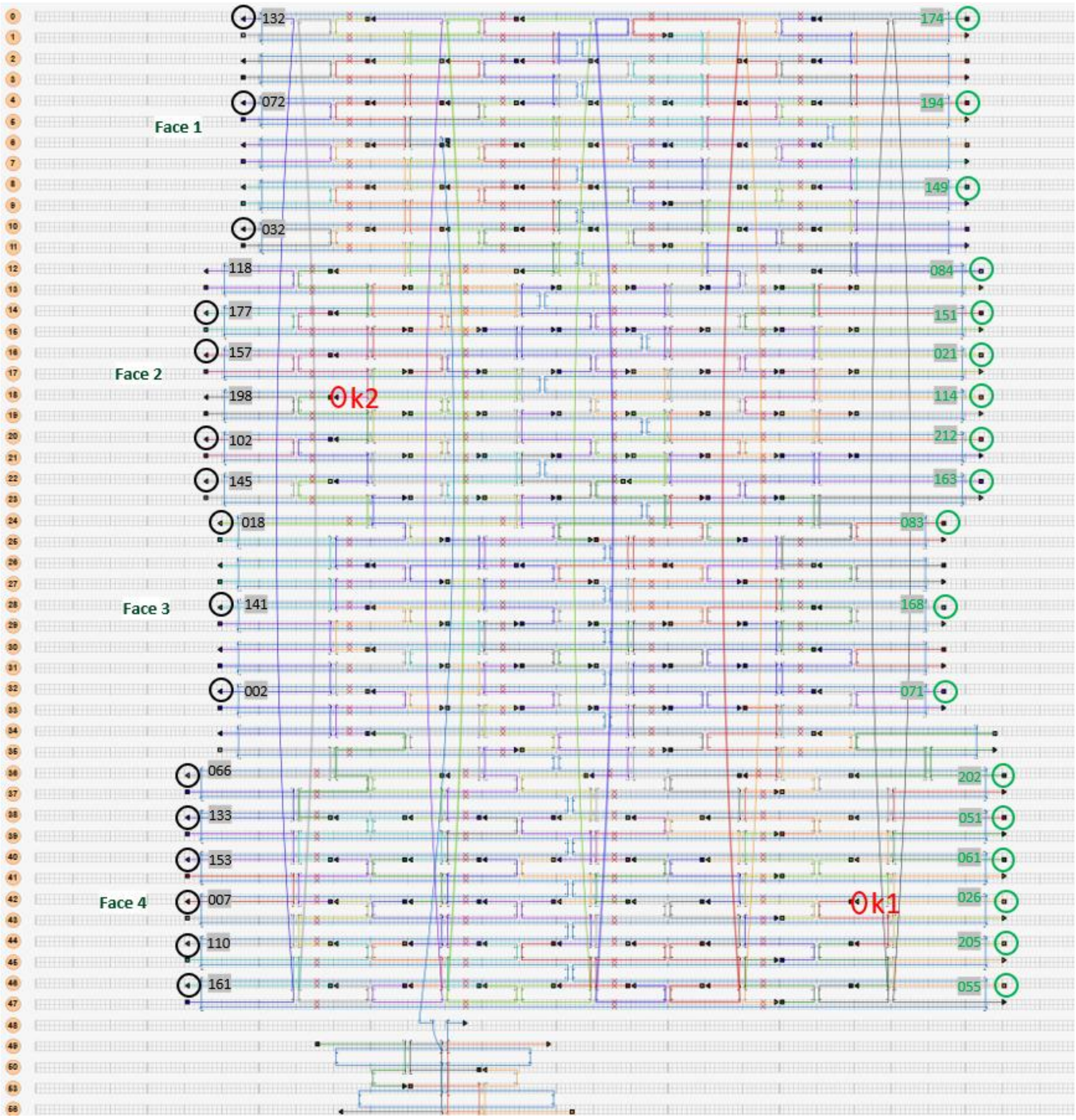

Figure s4 | Explanation of the box's designs, by marking of the functional staples of the lids, upon the lids' caDNAno design. We will define the "up" lid and the "down" lid, which are differentiate of their special stales. ***Black*** *marks represent the "up" lid;* ***green*** *marks represent the "down" lid;* ***dark green*** *marks represent both lids. The faces of the capsule are mentioned. The staples' edges Surrounded by a circle (black or green) represent the exit of a steaky end from that edge of the stale, which create the connection to the "up" or "down" lid respectively with complement steaky ends from the capsule (Figure s3 - main manuscript). The serial number of each staple is written aside with the matching color (see Table s2, Table s3). The staples' edges Surrounded by a red circle represent that this is the edge of the steaky end for the spring connection for the DOCS. K1 is for "up" lid, k2 is for "down" lid.*

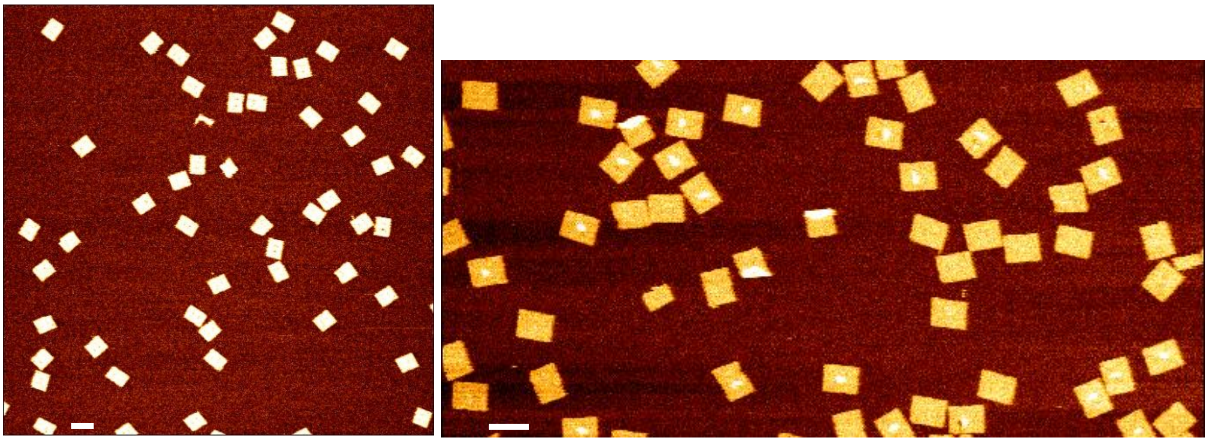

Figure s5| AFM images of the free "up" lid. Scale bars 100 nm.

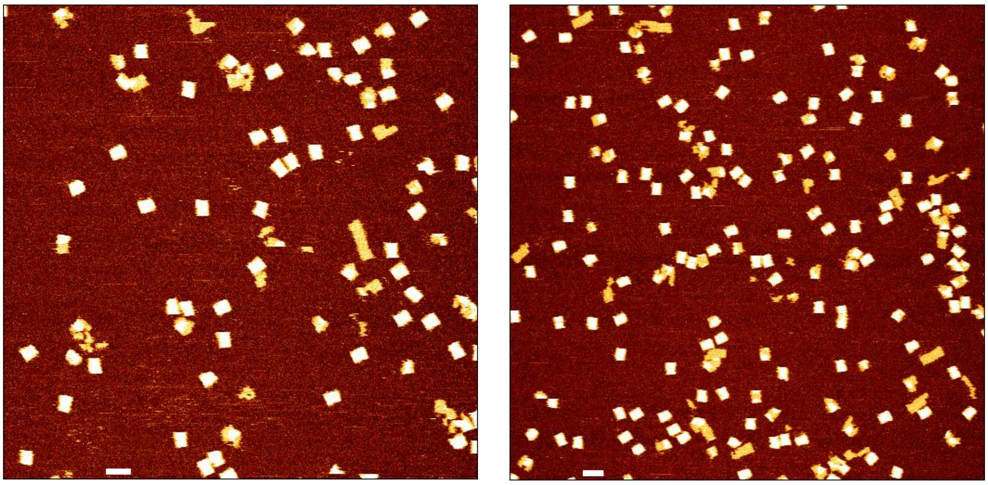

Figure s6| AFM images of the free boxes. Scale bars 100 nm.

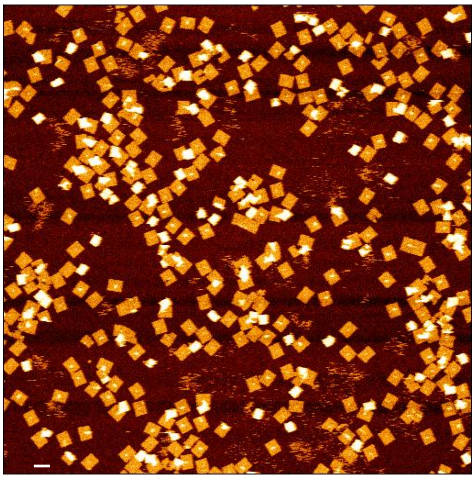

Figure s7| AFM IMAGES of the open capsule. Scale bar 100 nm.

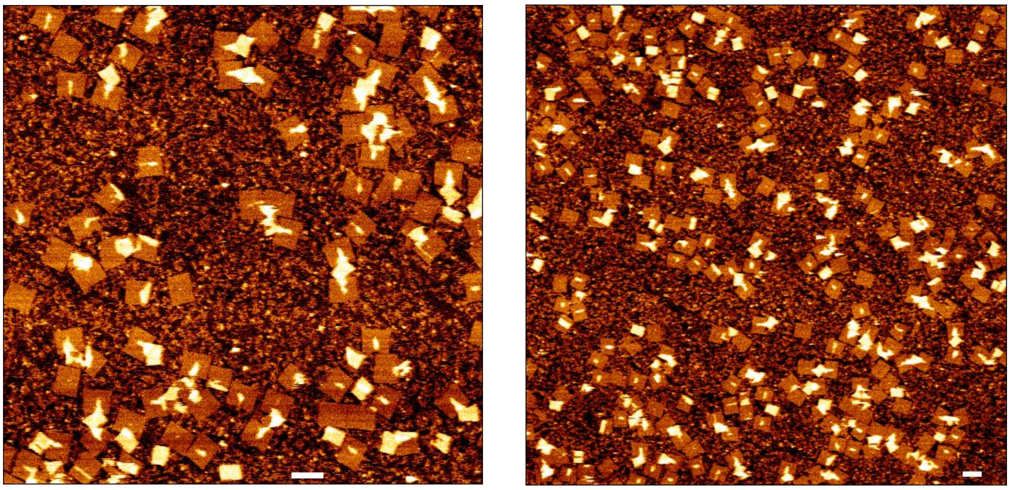

Figure s8| AFM IMAGES of the DOCS. Scale bar 100 nm.

Figure s9| See video "SNUPI model". A video of the DOC model performed by SNUPI software.

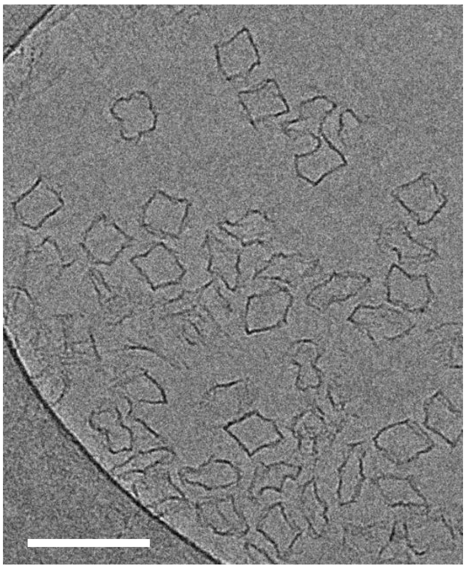

Figure s10| Cryo-TEM images of the free boxes, without the lids. The box structure without lids exhibited structural deformation compared to the closed ones, as shown in the cryo-TEM images (Figure s16), which is reasonable, due to the flexible nature of the lids and boxes which are made of single DNA layer. Scale bar 100 nm.

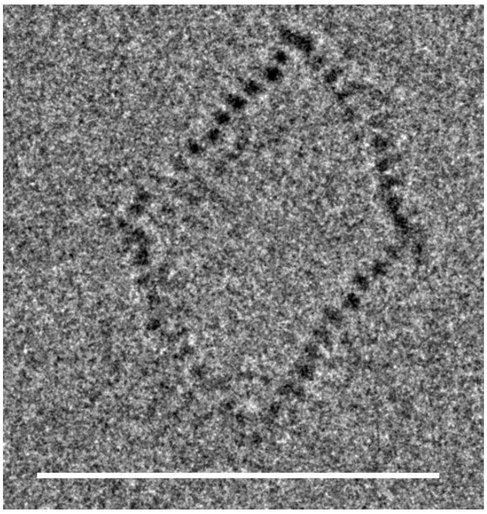

Figure s11| Cryo-TEM image of the one free box, without the lids. The parallel DNA-helixes of the can be seen. Scale bar 100 nm.

Figure s12| See video "only box tomo". Cryo-TEM tomogram video of the free box.

Figure s13|See video "DOC_2D". A Video of the DOC along z axis under the cryo-TEM.

Figure s14| See video "DOC_lids_box_1". A Video of DOC, together with free boxes and lids along z axis under the cryo-TEM.

Figure s15| See video "DOC_lids_box_2". A video of DOC, together with free boxes and lids along z axis under the cryo-TEM.

Figure s16|See video "DOC tomo with corrections". A video of the DOC's Cryo-TEM tomogram with hand corrections (see methods).

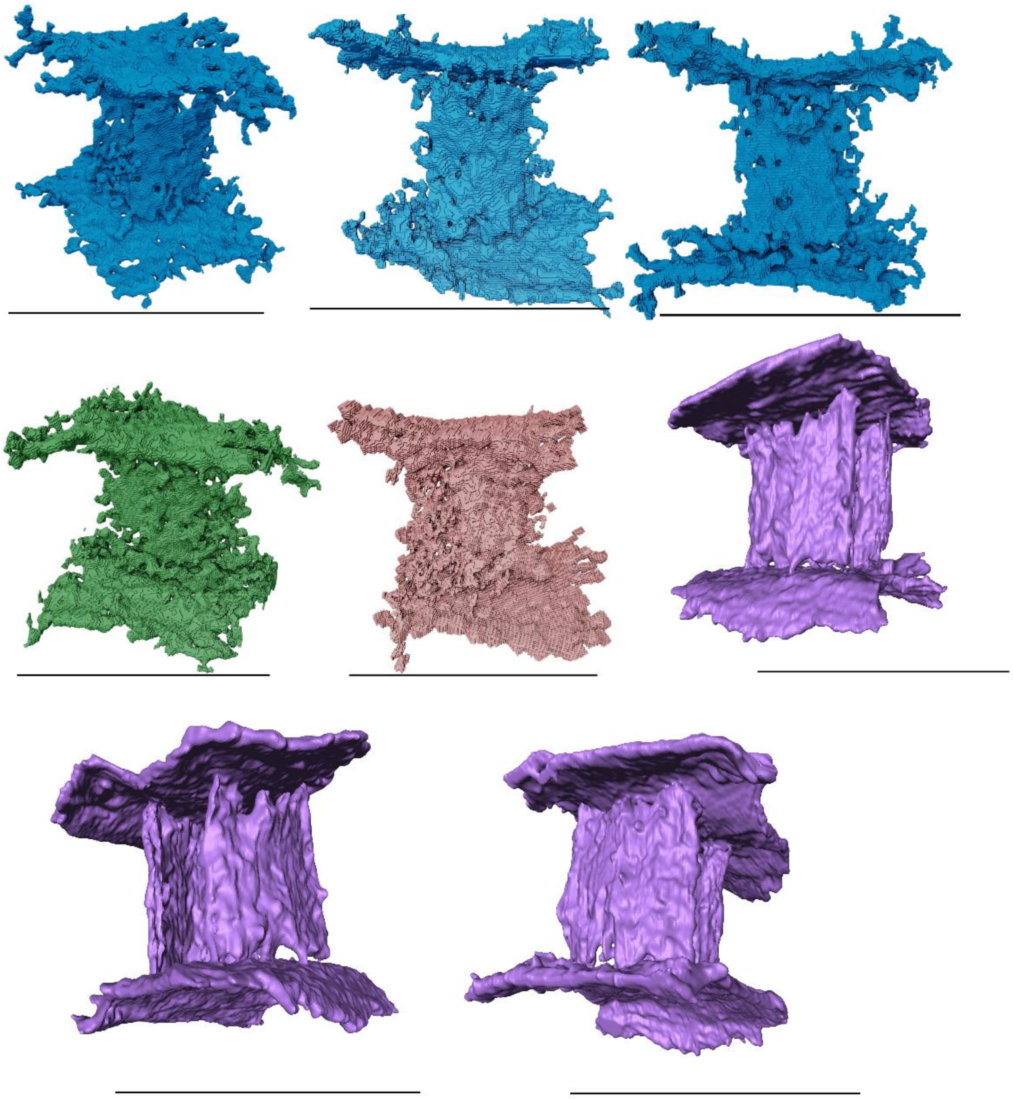

Figure s17| The DOC - cryo-TEM Tomograms. Scale bars 100 nm.

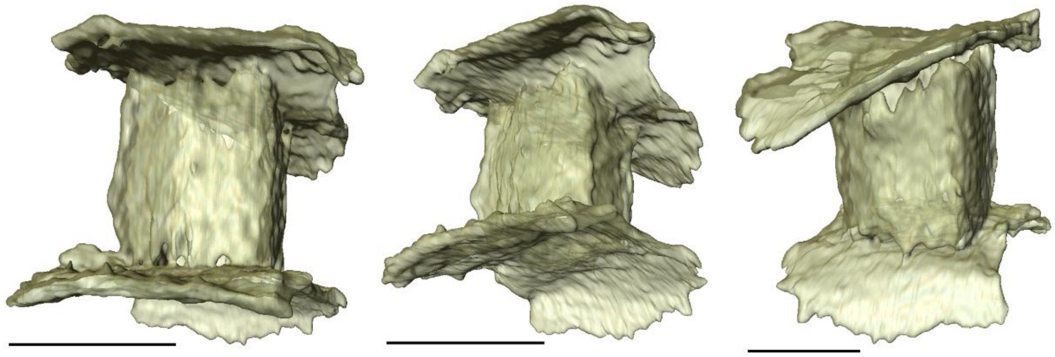

Figure s18| The DOC - cryo-TEM Tomograms (with hand correction, see methods). Scale bars 50 nm.

Figure s19| See video "DOC_2D". A video of the DOC along z axis under the cryo-TEM.

Figure s20| See video "DOC tomo with some corrections". A video of the DOC's Cryo-TEM tomogram with partial hand corrections (see methods).

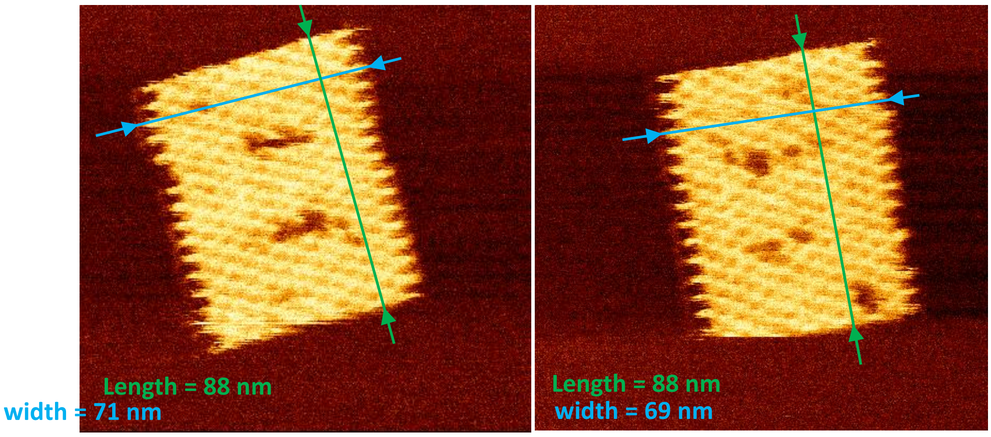

Figure s21| Examples of dimensions measurements of the lid, via AFM. The images also show defects holes that resulted from missing staples and were corrected for the final structure.

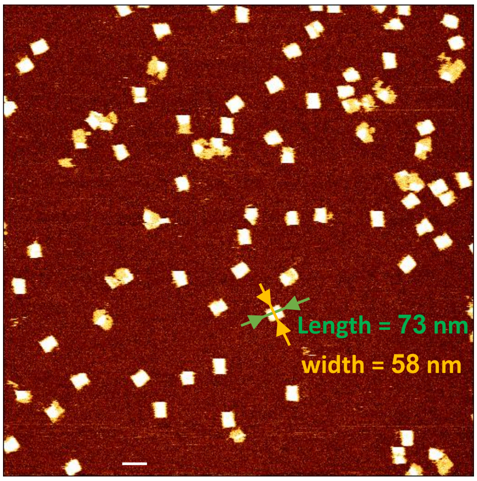

Figure s22| Example of dimensions measurements of the capsule, via AFM. Scale bar 100 nm.

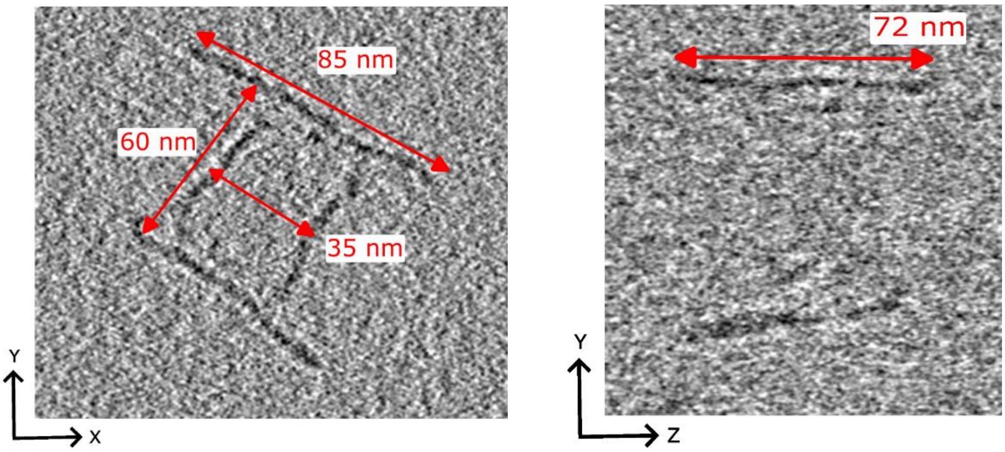

Figure s23| Example of dimensions measurements of the DOC (box and lids), via Cryo-TEM.

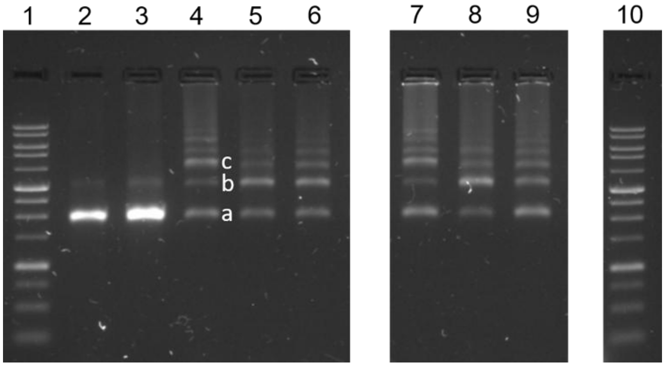

Figure s24| Gel electrophoresis of the DOCH. 1,10: ladder, 2: only the free up lid, which purified by AMICON 100K kit, 3: only the free down lid, which purified by PEG purification, 4: capsule with both lids (purified by AMICON 100K kit), 5,6: capsule with one lid only (purified by AMICON 100K kit), 7: capsule with both lids (purified by PEG), 8 ,9: capsule with one lid only (purified by PEG). All free boxes were purified by AMICON 100K kit. Explanation to 4: a - free boxes and lids, b - capsule with one lid only, c - the whole DOCH, bands above c - aggregates. It is visible that the binding of the components that were purified by AMICON are more efficient, when less aggregates are formed relative to the PEG purification. The bands corresponding to different the closed full capsule structure (DOCH) display relatively high intensity, suggesting efficient assembly. Their rather narrow width further indicates the uniformity of the structures. Notably, the results confirm the high efficiency of the hinges connections.

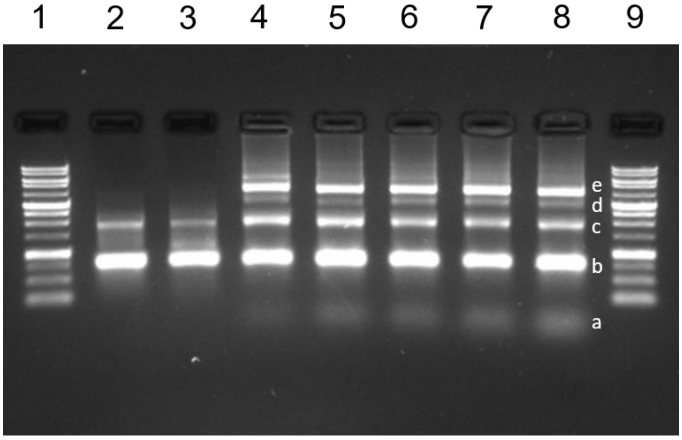

Figure s25 | Gel electrophoresis of the DOCS. 1,9: ladder, 2,3: only the free springs, 4: DOCS that were assembled without "blocker" and "anti-blocker" staples, 5,6,7,8: DOCS that were assembled with "blocker" and "anti-blocker" staples with concentration ratio of 1:1, 1:2, 1:3, 1:5 in correspond. explanation to 8: a – excess "blocker" and "anti-blocker" staples, boxes and lids, b – free springs, c – free boxes and lids, d – boxes with one lid only, e – complete DOCS, every band above e - aggregates. It is visible that the assembling manipulation using "blocker", and "anti-blocker" staples improved the assembling efficiency, when less aggregates are formed. Also 1:5 ratio exhibits the greatest efficiency. The bands corresponding to DOCS display relatively high intensity, suggesting efficient assembly. Their rather narrow width further indicates the uniformity of the structure. Notably, the results confirm the high efficiency of the hinges and springs connections. Furthermore, we gave a special attention to optimizing the spring's binding and employed a strategic approach to enhance its efficiency. First, we mixed the boxes with both lids, allowing them to bind exclusively through the hinges. Simultaneously, we introduced "blocker" staples, which partially bound to the box's sticky ends to facilitate controlled spring attachment. Next, the springs were added, able to bind only to the lid's sticky ends. Finally, we incorporated "anti-blocker" staples, which fully hybridized with the blocker, releasing the blocked sites and enabling the spring to connect to the box as well. When we cooled down the system, the capsules were closed while the spring's stem-loops were formed. This stepwise process ensured precise binding and improved efficiency (see Methods for further details). Capsules assembled without blockers or with insufficient blocker concentrations exhibited reduced efficiency, leading to the formation of more substructures and aggregates.

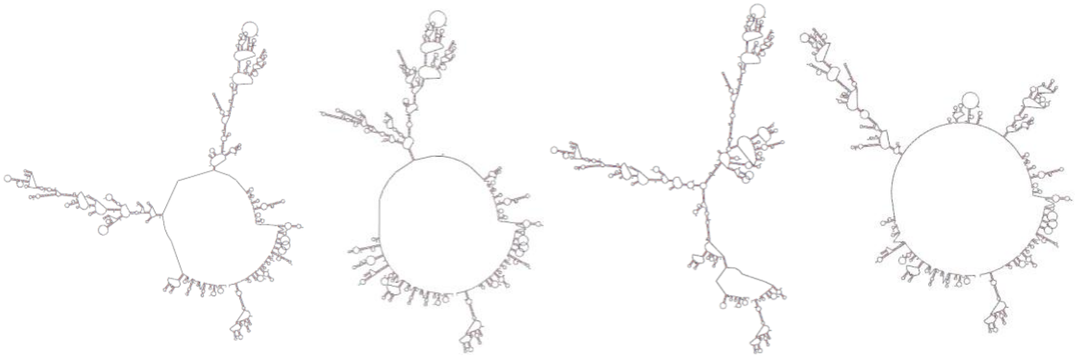

Figure s26| Examples of possible stem loops combination of the spring, performed by MFOLD, under the conditions of 25°C, 1.5mM Mg+2, 150mM Na+1.

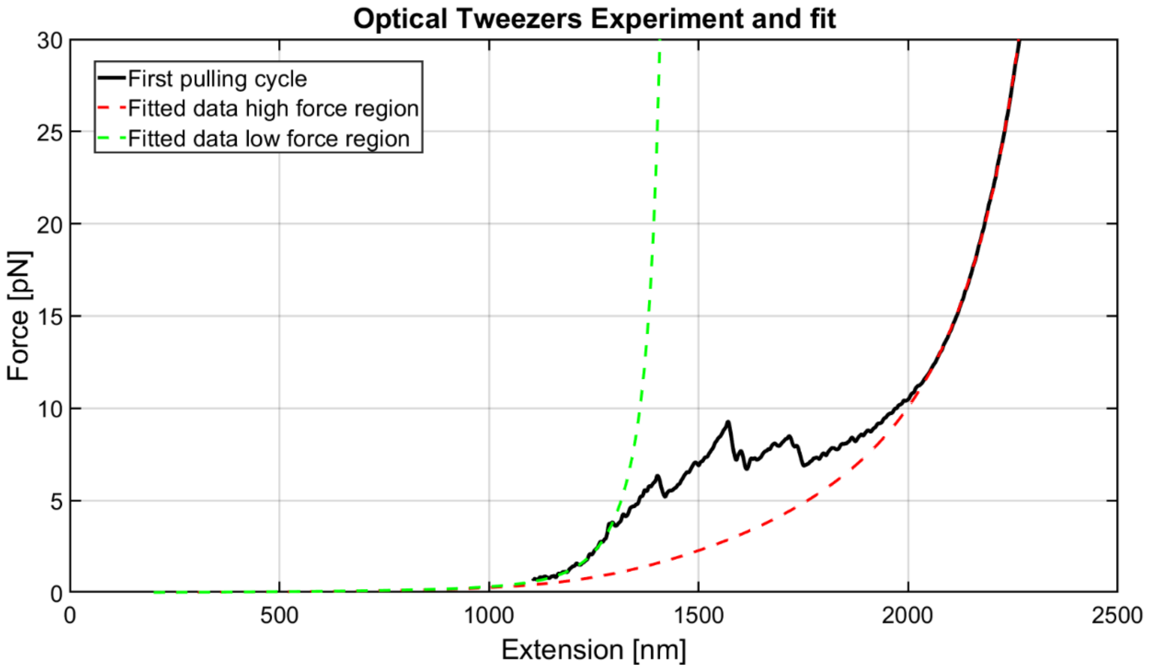

Figure s27| Force-extension curve of one spring, only the first pull. $L_p = 0.69$ *nm,* $L_0 = 108$ *nm.*

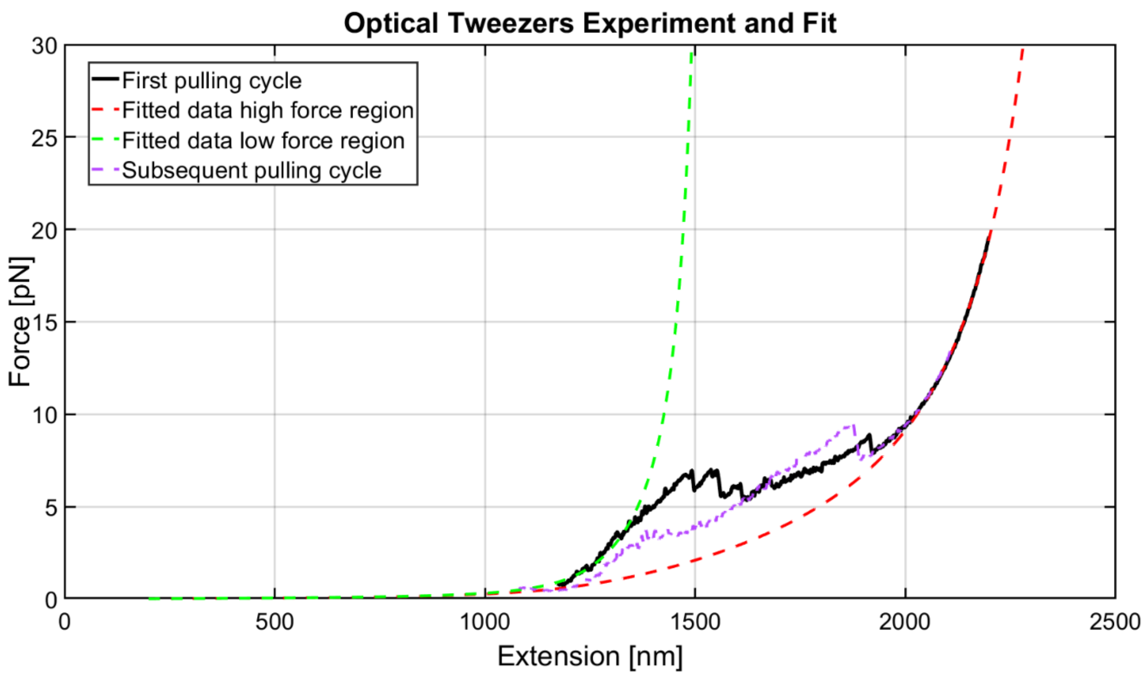

Figure s28| Force-extension curves of one spring. black – the first pull. purple – the additional pull. There was 30s dwell between pulls. Fit's parameters [nm]: $L_p = 0.77$, $\overline{L}_0 = 215$ *nm.*

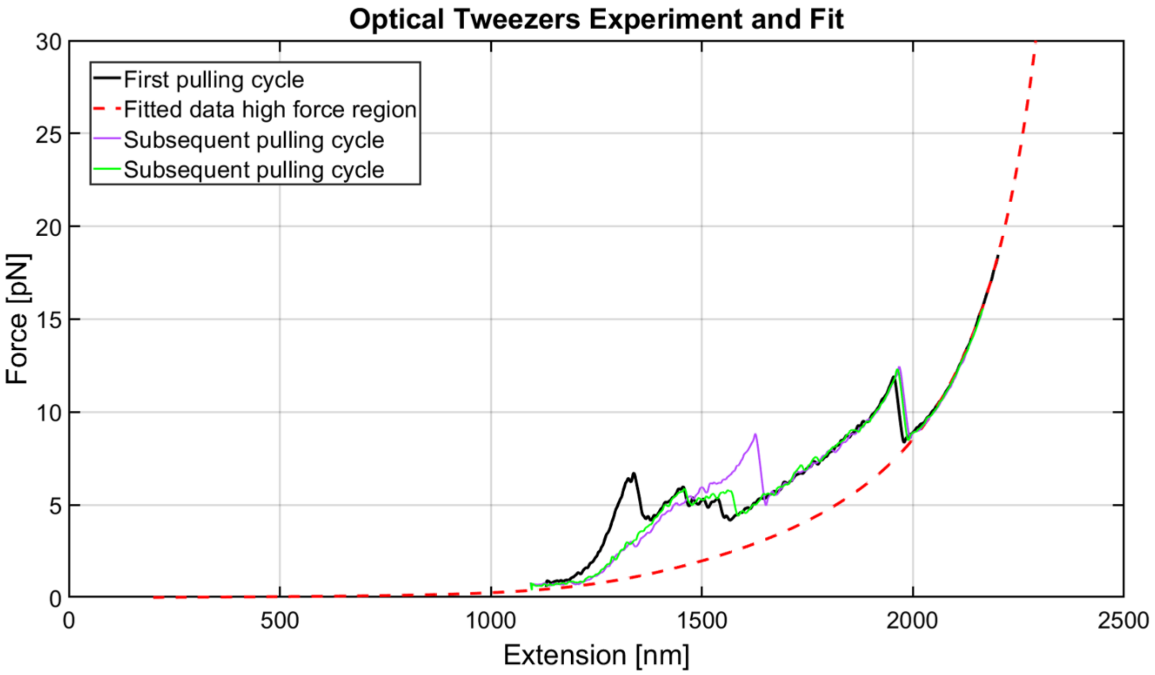

Figure s29 | Force-extension curves of one spring. black – the first pull. green – the additional pulls. There was 30s dwell between pulls. Fit's parameters [nm]: $L_p = 0.77$.

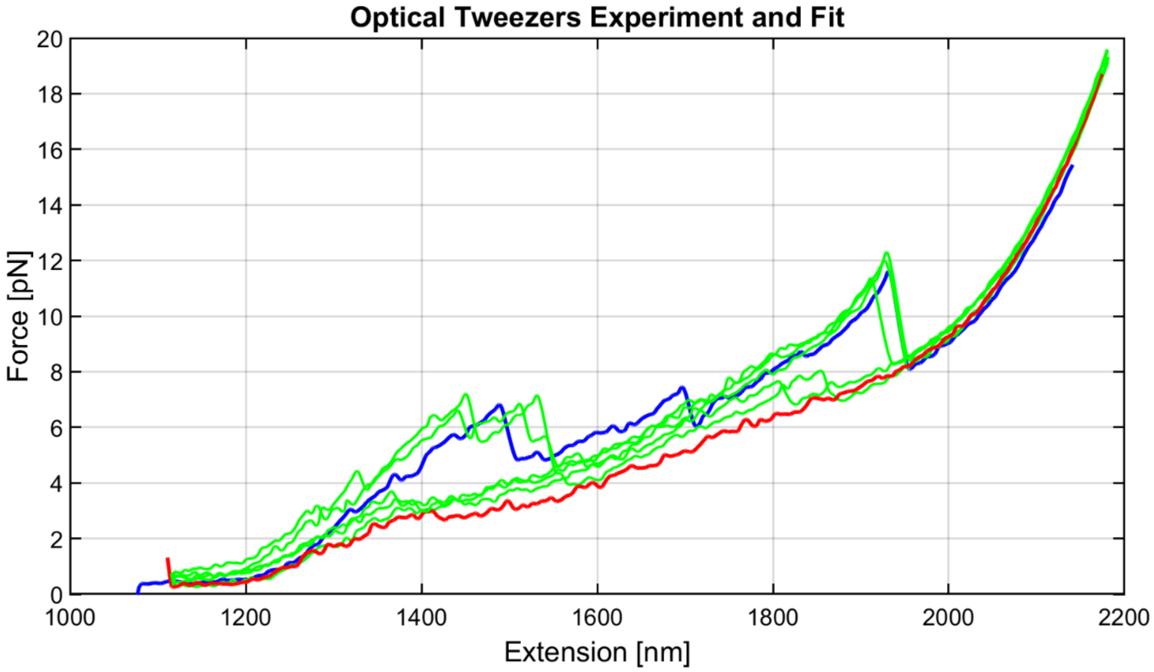

Figure s30| Successive force-extension curves of one spring. Green – several force-extension measurements of the same spring. purple – on of the last measurements, the loops do not form again. There was 30s dwell between stretches. 24 cycles were performed, only a few of the pulls are display, since many repetitions are needed for altering all the loops. Blue – the first cycle. Green – 5 of the 10 first cycles. red – one of the last 10 cycles.

Supplementary Tables

Name	Sequence (5' to 3')
cap_003	TCTAATCTGCACGAATATAGGGGAACAGGG
cap_004	GCAGATTCAAAAACGCTCATGGAATGCCTGAG
cap_005	TTTTTGGTGAATTATCACCGTGAAATTATTCATTAAATTTTT
cap_009	GCACGTAATCTGAATAA
cap_010	TAGCTGATGGTTGATAATCTTGTTGAGTGAGA
cap_012	AAGAGAGACCTCAAACGCGAGGCGCCGCCACC
cap_013	TGCGCCGATTGCAGGGAGTTAAATTCATGA
cap_015	TATCATAATACATAACGCCAAACCAGTCAGTCATTGTG
cap_019	TATAACGTTGCGGGAATATTCAACCGAGAGCGATTTTTC
cap_020	AGCTATATAGCATTAACATCCAATGTACCAAAAACATTATTATTTCA
cap_022	AACTTTAAGACGTTGGGAAGAAAAATCTACGCATTCAAC
cap_023	TCAGACGACATACATGAGTTTTAAGAAAGCGCAGTCTCTGAATTTTTT
cap_024	ATCCTAATAAGAACGGGTATTAAAAGCAAGCA
cap_025	TTTTTTGACTATTATAGTCAGAAGCAGAGAGTACCTTTAATTGCTCCTTTTT
cap_027	AAAAATCGCGCTCTATGCAACTAAGAGCTGAA
cap_028	AGTAATTCTTCGAGCCAGTAATAAGAGAATAACTAGAA
cap_030	AACGTTACGATGAACGGTAATCAGAGATCT
cap_031	AAGGCCGCGGCATTTTCGGTCACACCACCC
cap_033	CTTGATACTGAAAATCTCCAAAAAGGAGTGAG
cap_034	AATTACCTGGTTAGAACCTCAAGACTTGAACC
cap_035	ATCATTCCTTACGAGCATGTAGAAAGGTAA
cap_036	ACAAAGGCAAAAGGGTGAGAAATTTTAGAAAAAGCTAAATCGGTTAAATCATA
cap_037	TTTTTTTCTGGCCAACAGAGAAGTAATAAAAGGGACATTTTT
cap_038	ACCAATGATTTGGGAATTAGAGCCCCGATTGA
cap_039	TGGGTAATTACGCCAGCTGGCGGATCGCAC
cap_040	CAACCCGTGTAACCGTGCATCTGCAGCTTTCC
cap_041	AGATGGTTCCCAAATCAACGTAACCATAGGCT
cap_042	ATCGCAAGTACCTTTTTAACCTCTGAAAAC
cap_043	AATTCTTAAATGGTTTGAAATACAAATCCA
cap_044	CTCAGGAAAAAGGGGGATGCTCCGCATTTTGC
cap_045	CACAACAAAGCATAAAGTGTAAGGGCGCCA
cap_046	AACGAAATGACCCCCAGCGATTCATAAGGG
cap_047	TTTTTTAAATCCTCATTAAAGGATATTCAGAGGCAGG
cap_048	AAAGCCCCCCTTCCTGTAGTTTTCTTTTCCCA
cap_049	TATTTTTGGTAAAACTAGCATGTCGTATAAGC
cap_050	CTGAGCAACGCCTGATTGCTTTGTTGCGTA
cap_052	TAGAAGAACCATCACGCAAATTAAAAACAGGAGGCCGATTCGCTACA
cap_053	GTGGCAGAATCGCTCGAAATCCGATTATTT
cap_054	GGTCATAGACGTGGTGCTTGTTACAGCAACTC
cap_056	AGAGGTGTCACCTTGCTGAACCAGTTGGCA
cap_057	AATGTGAATAATTCGCGTCTGGAAAAACAG
cap_058	GAAGATTAATCATATGTACCCCAAATTAAT
cap_059	AAAACCAGATTTAGGAATACCATTAATAAATGGGCTTG
cap_060	ATTGACGCACCGACTTGAGCCAAACCATCG

cap_062	AATGACAACCAAAAATAAGAGCAAAAGACTCCTCAACCTTATGACAAGCATTTCA
cap_063	TTTTTGTTTGACCATTAGATACATTTGGCAAAGAATTAGCAAAATTATTTTT
cap_064	TTTTTAACACCCTGAACAAAGTCAGAAAAAGTAAGCAGATAGCCGAATTTTT
cap_065	AGGATTAAAGCGGATTGCATCAGAGAATGA
cap_067V2	*AGACTTCACCCCTCAA*ATGCTTTCCAATACTGCGGAATAGCAACAC
cap_068	GTAAATCGATTCGGTCGCTGAGGCCAATGACA
cap_069	GGGTGGTACCAGTGAGACGGGCGCCCGAGA
cap_070	TTTTTCAATCAATATCTGGTCTCAAATATCAAACCCTTTTTT
cap_071	GCCCAATCCAAGTACCGCACTCCTGAACAA
cap_073	TTCTGACGCCCTAAAACATCGCGATAAAAC
cap_074	AATGCCACGAACCAATCGGCTGTCGGTATTCT
cap_075	GGGAGGGAATCAATAGAAAATTCACGTAGAAAGCACGACTTAAGTGTGATTAAGT
cap_076	GTCGGTGGATTTACGCTCGCCCTGAGGAAACCGAGGAAAC
cap_077	AGAGATAACAATAGCTATCTTACCTAACGGAACCCCGCT
cap_078	CTCCAACGTCGAGGTGCCGTAAAAAGGAAG
cap_079	AGTCTGTCTCAAACTATCGGCCAGCCATTG
cap_080	AAGTGATTTTCTTGTGGCATCAATTGCTTTCCTCGTTAGACGAGCACG
cap_081	GAGCCGTCAGTTGAAAGGAATTGATGAAAAAT
cap_082	GTCAGTGCTGTAGCGCGTTTTCATGAAACGTC
cap_085	CCTCAGAGATAATCAAAATCACCGTTATTTAT
cap_086	CATATGCGGAGGCATTTGTCCAGACGACGACAAATATCCC
cap_087	ATTCATTTAAACAGTACATAAATCTTTCCCTT
cap_089	AACGGAGAACTTAGCCGGAACGATTCATCA
cap_090	ACAACCATAATAAACACCGGAATAATTGCGAA
cap_091	ATATCAAACGACCTGCTCCATGTTTTTGTATC
cap_092	ATCGCCTGATAACCTTGCTAAACGCCATTTCT
cap_093	CACCGGATTGAAGCCTTAAATCATTACCGC
cap_094	AGTAAATTTCAACAGTTTCAGCAAAGGCTC
cap_095	CAACAGGACCAGTCACACGACCTAGAACCC
cap_096	ACGCAAGGATAAAAATGGCCGGAGACAGTCAAAGGGTAGC
cap_097	TCCAGCCCAGTTTGAGGGGACGCAACATTA
cap_098	TAATTAATAATATATGTGAGTGAATAAATT
cap_099	GCCGCGCTGCGAAAGGAGCGGGCGATCAAGTT
cap_100	GCTTTCATACGACAGTATCGCCGGTACGAGGC
cap_101	TAAAGAAAAATACCAAGTTACAAAGGCGAATT
cap_103	TTTTTGAGTGTACTGGTAATAGCTTTTGACCGTTCCA
cap_104	ATAGCAGCCTTTAGCGTCAGACCTTGAGTA
cap_105	TGGAAGTATGCGATTTTTTAATAATTAAGAACTGGCTCATTATAAGGAATTA
cap_106V2	TGAAATAGCCCACAAGAATTGAGAAATAGC*AGCCTTTAAAATAAGA*
cap_107	TGAATCGGACATTAATTGCGTTGCGTAATCAT
cap_108	AGAATCCTCGGCTTAGGTTGGGTTATGTAAAT
cap_109	CCGGAAAGTAGATGGGCGCATCCGGATTCT
cap_111V2	*TAGTCTTTCACCGCCT*GCAACAGAGAGCCAGCAGCAAAGGAAGGTT
cap_112	GGCACCGCGCGGGCCTCTTCGCTACGCCAGGG

cap_113	GGGCGCGTACTATGGTGGCGAACGTGGCGAGAGCACTAAA
cap_115	GTAACATTTTGTTTGGATTATACTAACAGAAA
cap_117	TTTTTACCCATGTACCGTAACAGCAAGCCCAATAGGATTTTT
cap_119	TTTTTGTGTTTTTATAATCAGTGAGGCCACCGAGGAACGGTACGCCAGCGTAACCACCACACCC
cap_120	CTGGCCCTTCGGCAAAATCCCTTACGTGGA
cap_122	TTTTCCCAGGTTGTGAATTCATGCATACATACATAAAGGT
cap_123	CCGTGGGCATTTTTTAACCAATAAATTGTA
cap_124	CCACCACCGTACTCAGGAGGTTTAGGGGTTTT
cap_125	TCATAATTTAAAGCCCCAGACAGTACCGACAAAACCAATC
cap_126	CATAAATAACTCTGACCTCCTGCAATTCCA
cap_127	CTCAGAATTTTAGCGAACCTCCATAGAAGGCTTATCCTTTCCTT
cap_128	GGCTGACCGGCGCAGACGGTCAATATACCAAG
cap_129	CGCGAAACGCCACTACGAAGGCACGAGGACTA
cap_130	CCCAATCCCAGAGAGAATAACATAAATATCAG
cap_131	GTTTTGAATATTTTTAATTCGAGCCATGTTTT
cap_134	CCGCCACGCCTGTAGCATTCCATCATAGTT
cap_135	TTTTTTGCCCCCTGCCTATTTCCGTATAAACAGTTAATTTTT
cap_136	GATTTTCATCAATATAATCCTGAATCATTT
cap_137	GCAATAAGAAGCCCTTTTTAAGGGGTAATT
cap_138	AAATATTTAGGAACGCCATCAAAAGCGAGTAA
cap_139	AAGACTTTGGCCGCTTTTGCGGGATTAAACAG
cap_140V2	*CGGGAGCTCCGTTGTA*GCAATACAGTAATA
cap_142	CAAAAGGTTCGAGGTGAATTTCTCGTCACC
cap_143	AAAGCCTGAAGGCGTTAAATAAGCGCCCAC
cap_144V2	GCCGGAGATCACCATCAATATGGAAGCCTTGACCCTG
cap_146	GAGCGCTAAAACAGGGAAGCGCAAATAAAC
cap_147	ACAGTGCCGGAACCTATTATTCCCAGGCGG
cap_148	TTTTTGTAGAAAGATTCATCAGTTGAAAATAGCGAGAGGCTTTTGCATTTTT
cap_152	GCTGATGCCGACCGTGTGATAAATTTTAGTAT
cap_154	CTAAAGCAAGGCGGTCAGTATTAAAATGCGCG
cap_155	CATTCAAAGCAAAATCACCAGTCCATTAGC
cap_156	AATCAACAATAGATAATACATTATTCGACAAAAGAAACCACCAGAAGGAGCGGAATTATCATTTTTT
cap_158	TAATGCAGACCCTCGTTTACCAGATTAGACTG
cap_159	TGCGTATTAGCCTGGGGTGCCTAAAATTGTTA
cap_160	CAGGCAACGCAAATGGTCAATAACAGTTGA
cap_162	GGCAACAGTTTATTTTGTCACAAGGTAAAT
cap_164	AATCAGATCGACTTGCGGGAGGTTACCGCCTC
cap_165	AACTGATACTGAAAGCGTAAGAATTTACATTG
cap_166	TAGGGTTCCAGTTTGGAACAAGCTAAAGGG
cap_167	AAGAGGAAACCGGAAGCAAACTCCCTTAATTG
cap_169	ATAAGTGAATAGGTGTATCACCCTCATTTT
cap_170	TCCGCTCAGTTGGTGTAATGAGTACCTTGAAT
cap_171	TGCAAGGCCCTTAGTGCTGAATTGTTATTACGCAGTATGT
cap_172	GAAAAATATAAACAACATGTTCAACGCCAA

cap_173	CATGTAATTTAGGCATTATACA
cap_175	CCATAAAGTAATAGTAAAATGTCGACGATA
cap_176	GCTCAGTATGAAACATGAAAGTATCGCCACCA
cap_178	TGCGGAACACTCGTATTAAATCCTTTGCCCGATAGATTA
cap_179	CTGAATATAGTTTCATTCCATATAACCTGTTT
cap_180	CGATTTAGATATCGGCATAGTAAGCGTCATAA
cap_181	AGAGTAATACCGGATATTCATTATAATTTC
cap_182	GATAGCGTAAACAGTTCAGAAAACAAAAGATT
cap_183	AGCCATAGAACCAGAGCTACCAAATCTCAC
cap_184	CTCAGAGCTAGCCCCCTTATTAGCTTTTGTTT
cap_185	GCTAACTCCCAACGCGCGGGGAGTCACCGC
cap_186	TTCCCAAGCGGATGGCTTAGAGAACAGGTC
cap_187	AGCGTAACGATCTAAGGAACAACTAAAGGAATAATTTTTTCACGTCGATAGT
cap_188	CTTAAGCTCTGTTTCCTGTGTGATGAGTGA
cap_189	CAGGGATACTGAGTTTCGTCACCGTCTTTCCAGACGTT
cap_190	TTTTTAGACACCACGGAATAATATAAAAGGATGTTCTTCTAAGTGTCACGAC
cap_191	AACCATTAAGCACGTCAAAAATGATTAAGCCC
cap_192	GGAAGTTTGGTAAAATACGTAATAAAGTAC
cap_193	GTTGTAAGGAAGGGCGATCGGTTTCTGGTG
cap_195	AAAGAATAAACAGCTGATTGCCCTAGGCGGTT
cap_196	TTTTTCCCAGCTACAATTTTATCCTGACGCTAACGAGCGTCTTTCCATTTTT
cap_197	ATACGCTGTGCCATTCATTGAATCAATATCGC
cap_199	GTAAGCGTTTGGCCTTCCAGAATGCCGCCAGCATTGACAGGAGTTTTT
cap_200	AATAGAAAAGTTTTGTCAGTACAAACTACAACCCTCAGAG
cap_201	TCGGAACCAGTCCACTATTAAAGAATAAATCA
cap_203V2	AGCCCCCTTGACGGGGAAAGCCTGCTTTGAATCAGAG
cap_204	CGGCTGACTGTCCCGCCAAAATAATACCCAAAAGAACTGGCATGATTGAAACAA
cap_206	TTTTGGGGTCAAAGGGCGAAAAACGATGGTGG
cap_207	ATAGCGATTACCTTTTTTAATGGCAATTAC
cap_208	GCAACTATATATATAACCGATATTCGCTAT
cap_209	TTTTTTTATCAACAATAGATAAGTCATCGAGAACAAGCAAGCCGTTTTTTT
cap_210	AACCGAATGTACAGACCAGGCGAAAGCTGC
cap_211	GGAAGAAATAATGCGCAAAGGGATTTTAGACAGTAAAAG
cap_213	TCAGAGCTAAGAGGCTGAGACTAGGATTAG
cap_214	ATCTAAAGAGCACTAACAACTAAACGTTATAAGTTTGA
cap_300	AAGCCAACGCTCAACAGTAGGGCTTTTTT
cap_301	CGCGAGAAAACTTTTTCAAATATATTTTT
cap_302	TTAAGACGCTGAGAAGAGTCAATATTTTT
cap_303	GATGAAACAAACATCAAGAAAACATTTTT
cap_304	GTCAGATGAATATACAGTAACAGTTTTTT
cap_029	TTCCGAAAGAGAGAGTTGCAGCAATGCATTAA
cap_121	CTCAGCAGGCTACAGAGGCTTTCAACCTAA
cap_116V2	AACGATTGTTTGCCATCTTTTCCCGCCACC
cap_150	GATTAGCGTACCGCCACCCTCACCTCAGAA

cap_006	TAGCAAATATGGTTTACCAGCGAAGGGCGA
cap_088V2	*TAGCTCAATTCAAAGC*GAACCAGGCCCGAA
cap_008V2	*TGGGGCGCAGTACGGT*GTCTGGAAATGCTG
cap_014V2	*TAATACTTTCTACTAA*TAGTAGTTTTCATT
cap_016V2	*TGGATTATACGTGGCA*CAGACAATGGCTAT
cap_011V2	*ACATCACTATACCTACA*TTTTGATCTGAAA

Table s1 | Native staple strands of the box.

Name	Sequence (5' to 3')
cap_132_SE	TTTTTTTCTCAGGAGAAGCCAGGGTGAAACGCAATTTTT**CTGCTGTTGGTCGCAATC**
cap_072_SE	TTTTTGACAGAATCAAGTTTGCACCGTAATCAGTAGCTTTTT**GATGCGAAGTAGGCACTC**
cap_032_SE	TTTTTTATAAGTATAGCCCGGCCGTCGAGAGGGTTGATTTTT**AGCAGTCGCAGTGTCTGA**
cap_118_SE	TTTTTATTTTGCTAAACAACTGAATTTTCTGTATGGGTTTT**GGATGCGTCGGAAAAGC**
cap_177_SE	TTTTTGGTTTATCAGCTTGCTAGCCTTTAATTGTATCTTT**CACTGGATGTGACCCAAA**
cap_157_SE	TTTTTGAACGAGGGTAGCAACGCGAAAGACAGCATCGTTT**GGGTAAGGTAGGGTCACT**
cap_198_SE	TTTTTCTAAAACACTCATCTTGAGGCAAAAGAATACATTT**TTGTCCGGGTTTGTGGAA**
cap_102_SE	TTTTTGAGGACAGATGAACGGCTGACCAACTTTGAAATTT**ACTACTCTGGCTAACCCG**
cap_145_SE	TTTTTCTGACGAGAAACACCAGTGAATAAGGCTTGCCTTT**AGTGCGCCTTCGAGTTGA**
cap_018_SE	TCATTCAGAACGAGTAGTAAATACGAACTAACGGAACAACATTATTACAGTTTT**GTACTAGTCGTGGCGCTA**
cap_141_SE	TTTTTAAAGAAGTTTTGCCAGAGGGGTCAAAAATCAGGTCTTTACCCTTTT**GTCAGTGGAGTGGATGAA**
cap_002_SE	TTTTTTTTTGATAAGAGGTCATTTTTTTCTGCGAACGAGTAGATTTATTTT**ATGCAAGTGCACACATGG**
cap_066_SE	TTTTTAGCAATAAAGCCTCAGAGCATCCCTCATATATTTTAAATGCAATGCCTGTT**CAGAGTTTACGTCGCCTA**
cap_133_SE	TTTTTAGTAATGTGTAGGTAAAGATTCTATCAGGTCATTGCCTGAGATT**CTAAGCGTGTCGATGGGA**
cap_153_SE	TTTTTGTCTGGAGCAAACAAGAGAATATATTTTGTTAAAATTCGCATTT**GCTAACGCAGACTAGACG**
cap_007_SE	TTTTTTAAATTTTTGTTAAATCAGCTAACAAACGGCGGATTGACCGTTT**TACGCGTTCCAAGTGTGG**
cap_110_SE	TTTTTAATGGGATAGGTCACGTTGGTCCAGGCAAAGCGCCATTCGCCTT**AACTACTGTGGATACGCG**
cap_161_SE	TTTTTATTCAGGCTGCGCAACTGTTGAACGACGGCCAGTGCCAAGCTTT**GACCTTGTCACGGATAGC**

Table s2 | Box's staples for hinges conjugations with the "up" lid.

Name	Sequence (5' to 3')
cap_174_SE	**ATATGGTGAAGCGTACGG**TTTTCAAAGTTACCAGAGAGTGACTCTATGATACCGACTTTTT
cap_194_SE	**TATACCCGACCCTGGTGT**TTTTGAGCCTAATTTGCCAGTTACAATTAGACGGGAGAATTAACTGTTTTT
cap_149_SE	**GCGATGCCCTCTCTTACA**TTTTTTTATTTTCATCGTAGGAATCAAGATTAGTTGCTATTTTGCATTTTT
cap_084_SE	**AAGAGGATGTCGGCTCCT**TTTAATTGAGAATCGCCATATTTAACAGCTAATGCAGAACGCGCCTGTTTTT
cap_151_SE	**GATCCAAGCACTGCTAAG**TTTTTTAGTTAATTTCATCTTCTGACCTAAATTTCCAGTATA
cap_021_SE	**ATCCCTAGAGCACACAGT**TTGTGAATTTATCAAAATCATAGGTCTGAGAGACACAAAGAA
cap_114_SE	**GCGACCACCTAGTACAGT**TTAAATTAATTACATTTAACAATTTCATTTGAATAGCTTAGA
cap_212_SE	**CTGAACTCTCCGCCCATT**TTACCTTTTACATCGGGAGAAACAATAACGGATTAAGAAGAT
cap_163_SE	**CAGGTAGGCGTAGAGGTT**TTCATATTCCTGATTATCAGATGATGGCAATTCAGGTTTAAC
cap_083_SE	**CAACAATCTGGGTCCTAG**TTTTTTTAGACTTTACAAACATGAGGATTTAGAAGTATTTTT
cap_168_SE	**TGTAGGATCCGATCTCGG**TTTTTCGAACCACCAGCAGAACATTAAAAATACCGAATTTTT
cap_017_SE	**GCGTATGCACAACACAGA**TTTTTGAACAATATTACCGCCTTGCTGGTAATATCCATTTTT
cap_202_SE	**AGGACGGAAGCTATGCCA**TTCGGTCACGCTGCGAATCCTGAGAATTTTT
cap_051_SE	**ACCTGTGCTTGATCCTGA**TTACTACGTGAACCATCACCCAACTAGGGCGCTGGCAAGTGTAGTTTTT

cap_061_SE	**AGTGATGGCCTGACAGTG**TTCAGCAGGCGAAAATCCTGTTTCGTCTATCAGGGCGATGGCCCTTTTT
cap_026_SE	**ATGGCACTAGGCACAAGG**TTCGGGAAACCTGTCGTGCCAGCGCGGTCCACGCTGGTTTGCCCTTTTT
cap_205_SE	**TAACCGGTGTGTGAGGCA**TTCCGGGTACCGAGCTCGAATTCGCTCACTGCCCGCTTTCCAGTTTTTT
cap_055_SE	**GGACAAGCCGGGATTTGA**TTAGTGCGGCCCTGCCATCTGTACTCGATAAAGACGGAGGATCCTTTTT

Table s3| Box's staples for hinges conjugations with the "down" lid.

**Name**	**Sequence (5' to 3')**
cap_132	TTTTTTTCTCAGGAGAAGCCAGGGTGAAACGCAATTTTT
cap_072	TTTTTGACAGAATCAAGTTTGCACCGTAATCAGTAGCTTTTT
cap_032	TTTTTTATAAGTATAGCCCGGCCGTCGAGAGGGTTGATTTTT
cap_018	TCATTCAGAACGAGTAGTAAATACGAACTAACGGAACAACATTATTACAGTTTTT
cap_141	TTTTTAAAGAAGTTTTGCCAGAGGGGTCAAAAATCAGGTCTTTACCCTTTTT
cap_002	TTTTTTTTTGATAAGAGGTCATTTTTTTCTGCGAACGAGTAGATTTATTTTT
cap_066	TTTTTAGCAATAAAGCCTCAGAGCATCCCTCATATATTTTAAATGCAATGCCTGTTTTT
cap_133	TTTTTAGTAATGTGTAGGTAAAGATTCTATCAGGTCATTGCCTGAGATTTTT
cap_153	TTTTTGTCTGGAGCAAACAAGAGAATATATTTTGTTAAAATTCGCATTTTTT
cap_007	TTTTTTAAATTTTTGTTAAATCAGCTAACAAACGGCGGATTGACCGTTTTTT
cap_110	TTTTTAATGGGATAGGTCACGTTGGTCCAGGCAAAGCGCCATTCGCCTTTTT
cap_161	TTTTTATTCAGGCTGCGCAACTGTTGAACGACGGCCAGTGCCAAGCTTTTTT

Table s4 | Box's staples for no hinge's conjugations with the "up" lid.

**Name**	**Sequence (5' to 3')**
cap_174	TTTTTCAAAGTTACCAGAGAGTGACTCTATGATACCGACTTTTT
cap_194	TTTTTGAGCCTAATTTGCCAGTTACAATTAGACGGGAGAATTAACTGTTTTT
cap_149	TTTTTTTTATTTTCATCGTAGGAATCAAGATTAGTTGCTATTTTGCATTTTT
cap_084	TTTTTTAATTGAGAATCGCCATATTTAACAGCTAATGCAGAACGCGCCTGTTTTT
cap_151V2	TTTTTTTTTAGTTAATTTCATCTTCTGACCTAAATTTCCAGTATA
cap_021	TTTTTGTGAATTTATCAAAATCATAGGTCTGAGAGACACAAAGAA
cap_114	TTTTTAAATTAATTACATTTAACAATTTCATTTGAATAGCTTAGA
cap_212	TTTTTACCTTTTACATCGGGAGAAACAATAACGGATTAAGAAGAT
cap_163	TTTTTCATATTCCTGATTATCAGATGATGGCAATTCAGGTTTAAC
cap_083	TTTTTTTAGACTTTACAAACATGAGGATTTAGAAGTATTTTT
cap_168	TTTTTCGAACCACCAGCAGAACATTAAAAATACCGAATTTTT
cap_017	TTTTTGAACAATATTACCGCCTTGCTGGTAATATCCATTTTT

Table s5 | Box's staples for no hinge's conjugations with the "down" lid.

**Name**	**Sequence (5' to 3')**
cap_029_vavK1	TTCCGAAAGAGAGAGTTGCAGCAATGCATTAATTTATGGCCTAGACGGCGAGCCTGGGTT
cap_121_vavK2	CTCAGCAGGCTACAGAGGCTTTCAACCTAATTTATGGCCTAGACGGCGAGCCTGGGTT
Blocker	AACCCAGGCTCGCCGTCTAGGCCATGTATCCTTCGTCGAACGA
Antiblocker	TCGTTCGACGAAGGATACATGGCCTA

Table s6 | Box's staples for spring conjugations.

**Name**	**Sequence (5' to 3')**
Lid_002	GAGGGGACCGAGCCAGTAATCAGCTTAAATAA
Lid_003	TTTTTTAAAACGAACTAACGGGAAAAATCTACGTTAATTTTT

Lid_004	TCATCGCCGGAAGTTTCCATTAAAGCTTGCAG
Lid_005	CCGTAATGGCCTTCCTGTAGCCAGGTATAAGC
Lid_006	TTTCCAGTGCTAAACAACTTTCGAGGTGAA
Lid_009	AGTATTAAGCGAAAGACAGCATGTAGCAAC
Lid_010	CAGCCATTTCAGTGAGGCCACCGATAAAGGGA
Lid_011	TCACACGATATTAGTCTTTAATGCAGAGCCAG
Lid_012	GGAGTTAAGGCTCCAAAAGGAGCCACAACTAA
Lid_013	CAATACTTAGAAGAACTCAAACTATCGGCC
Lid_014	GTGTTGTAGGGAGCCCCCGATTGGGCGCTG
Lid_015	AACAGTAATAAACACCGGAATCAGAAAACT
Lid_016	ATTAAAGGGTGCCGTAAAGCACCGTAACCA
Lid_017	CCGCCTGTGGTTTGCCCCAGCATCAAAGGG
Lid_018	TCATTAAAGAACCTATTATTCTGACGGGGTTT
Lid_019	ATTGGGCATAAATCAAAAGAATAGGGTTGA
Lid_020	TCGACTCTTGCGTTGCGCTCACGTTTGCGT
Lid_021	TTTTTTAGAACCCTCATATATTTTAA
Lid_022	TTTTAGACTAGCGGTCACGCTGCGTAAATCGG
Lid_023	TGAATTATGTAGCGCGTTTTCATCAAATCACC
Lid_024	GAAATACCCGCCAACATGTAATTTGGTAAAGT
Lid_025	TTTTTAGCAAACTCCAACAGGAGCGAACCAGACCGGATTTTTT
Lid_026	TGGAAGGGACAAAATCGCGTGCATCCAGCCAG
Lid_027	TTCATGATGATAAATTGTGTCGCCTGCTCC
Lid_028	GAATCCTGGGAAACCTGTCGTGTAATGAAT
Lid_029	TATTTCGGCCAGAATGGAAAGCGGCCTTGA
Lid_030	GTGCCCGCCGTTCCAGTAAGCGTTGACAGG
Lid_031	CATTGCCCGCATTAAATTTTTGTCATTTTT
Lid_032	ATTTGCACGGATTCGCCTGATTGCCCTGAGCA
Lid_033	GATTCATCTGGCTCATTATACCAGCATCAAGA
Lid_034	CAAGTACCTAAATCAAGATTAGTTGCGTCTTT
Lid_035	TTGCTGGTCATCACGCAAATTAACAATCAGAG
Lid_036	TTTTTTGGCCCACTACGTGAACCGTCTATCAGGGCGATTTTT
Lid_037	TCAATCAGACAAGAACCGGATATGCGATTT
Lid_038	CTGACCTTTATCAAAATCATAGTGCTTCTG
Lid_039	CCGCCTGCAACAACTAATAAAAGGGCGAGGAT
Lid_040	CTGAATCTGACTATTATAGTCAGATTTAAACA
Lid_041	GGGGTCGAAACGTGGACTCCAACGGGCGAAAA
Lid_042	TGAATAAGAGCCCCCTTATCGCGAAAATCTAG
Lid_043	TTTTTAGTACCGCCACCCTCACGTACTCAGGAGGTTTTTTTT
Lid_044	ATAGCCCATTTACATTGGCAGATTCACCAG
Lid_045	ACAGGGATTATTTATCCCAATCGAGGCGTT
Lid_046	AATAGTAGGCTACAATTTTAATGCAATATATC
Lid_047	CCAATCATAATGCAGAACGCGCCAACGCTC
Lid_048	GCGCCCAAGGTATTCTAAGAACGCCAAATAAG
Lid_049	ACGCCTGCGAATAATAATTTTTAATCTCCA

Lid_050	AAACGATTCTTTACAGAGAGAATACCAATAAT
Lid_051	TTTTTGATGCAAATCCAATCGACTATATGTAAATGCTTTTTT
Lid_053	ATCGATGAAATTGTAAACGTTAAATAATTC
Lid_054	CAATAACGTAAAACAGAAATAAATTATCAT
Lid_056	TGTGTGAACGAGCCGGAAGCATGCCCTTCA
Lid_057	TTTTTGATTTAGTTTGACCATATTCTGCGAACGAGTATTTTT
Lid_058	GGAAACCCGACGGCCAGTGCCACCTGCAGG
Lid_059	GCATAGTATCCCCCTCAAATGCAGCAAAGC
Lid_062	ACGGAAATACATAAAGGTGGCAAAGGAAAC
Lid_063	TTTTTACATCACTTGCCTGAGTCTTTGATTAGTAATATTTTT
Lid_066	TTAGCGATTTTCATCGTAGGAATGTAGAAA
Lid_067	GCAAGTGAGGAACGGTACGCCAGAAGTGTT
Lid_068	AAAAAAAAGGCCGCTTTTGCGGAAGACTTT
Lid_069	CAATATATGATGAAACAAACATGGGAGAAA
Lid_070	TTTTTACGAGCACGTATAACGTACTATGGTTGCTTTGTTTTT
Lid_072	TCCTGTTTCGGGCAACAGCTGATTAAAGTGTA
Lid_073	TGTGTAGTAAATCGGTTGTACCACCTCAGAGAATTAGCAAAATTAAGTTTTT
Lid_074	TTTTTTTTGCCAGAGGGGGTACTTTTGCAAAAGAAGTTTTTT
Lid_075	AATACCTACCACCAGCAGACTGAGAATCAGA
Lid_076	CACCATTAACCATCGATAGCAGCAGCCACCCT
Lid_077	AAATATTTAACGGTAATCGTAAAAAATGGTCA
Lid_078	TTTAATCATGCAGATACATAACATTACGAG
Lid_079	GACGGGGAATTCATCAATAGAGATAGCCCTAA
Lid_080	GAACGAACATTTTGACGCTCAATCGTCTGA
Lid_081	ACATTCTGGCCAACAGATACGTGGCACAGACATCACCTTG
Lid_083	CAGCAAATGAAGGTTATCTAAAATTTTGAGGA
Lid_084	AATTCTGTAACGGGTATTAGGAGAATGCCAAC
Lid_085	AACATTACGTGGGAACAAACGGCGATCGGT
Lid_086	AGCGTCCAAGACGACGATAAAAACGGTAGAAA
Lid_087	CGGGAGCTCGCCGCGCTTAATGCGAGTTTTTT
Lid_088	CCAAGTTTTAGAACCTACCATAGAAGGAGC
Lid_089	CCAGAGCCCCTGAACAAAGAAAAAAAATCTCA
Lid_090	AATCCCTTGCCAGGGTGGTTTTTCAGCTAACT
Lid_092	TTTTTAGCTGGCGAAAGGGGGTCTTCGCTATTACGCCTTTTT
Lid_094	AACCCTAATCCAGTTTGGAACAAGATCGGCAA
Lid_095	TTTTTTACAGACCAGGCGCATGGACAGATGAACGGTGTTTTT
Lid_096	GCGTCTGGGATAGGTCACGTTGGCCATTCA
Lid_097	TTTTTATGCGTTATACAAATTGCCTGTTTAGTATCATTTTTT
Lid_098	ATTTTTGAAAGTACCGACAAAAAGGCAGAG
Lid_099	AGGAATTGTAGCATTCCACAGACAGCCCTCAT
Lid_100	TTTAGAAGAACAAAGAAACCACCATCAAAATT
Lid_101	CGAATTCGGTGCCTAATGAGTGTTTTCACC
Lid_102	TGTCTGGTTTAGCTATATTTTCACAAGAGA
Lid_103	TTTTCAATTTTAACCTCCGGCTACATAAAT

Lid_104	TTTATAAGCAACAGGAAAAACGCTCATGGA
Lid_105	TTGGGAAAACAACATTATTACACAAAATAG
Lid_107	TAGAGCCGTCATCATATTCCTGATCTGAATAA
Lid_108	TTTTTAAACGTCACCAATGAACCATTAGCAAGGCCGGTTTTT
Lid_109	GGATTGCAGAGCTTAATTGCTGTGTAGCTC
Lid_110	GTAATCTTTAAGGGAACCGAACTGTTATACCA
Lid_111	GTTATATACAAGACAAAGAACGCGATAATTAC
Lid_112	CGTTTGCCGAGGCTTTGAGGACTAGATCGTCA
Lid_113	GGAATTATCAATAGATAATACAATCTTTAG
Lid_114	TGAACACTAATTTGCCAGTTACGAGGTTTT
Lid_115	TAAGAACAGTTGAGATTTAGGATAACCCTC
Lid_116	TGGGAATTACAGAATCAAGTTTGCCGCCTCCC
Lid_117	TTTTTGATGAATATACAGTAATTCAGGTTTAACGTCATTTTT
Lid_119	TAAAACAGAAGGAGCGGGCGCTATAGAGCTT
Lid_120	GATGGCAAAGCCGGCGAACGTGAAGGGAAG
Lid_121	TCCTGATTCGCGCGGGGAGAGGCGTGCCCGCT
Lid_123	CGAGAGGATAGTAAAATGTTTATAATTCGA
Lid_124	AGTGAGAGATGGTGGTTCCGAAAGTCCACT
Lid_125	CGGTCATGCTTGCCCTGACGAGACGAGTAG
Lid_126	GGCTGCGCCAGGGTTTTCCCAGACCGAGCT
Lid_127	AAGCCTGGGTAATCATGGTCATAGATTAAGTT
Lid_128	CCACACCAAACAGGAGGCCGATGTAAAAGA
Lid_129	GGGTAACGCAACTGTTGGGAAGGGCGGATTGA
Lid_130	AGATAGCAGAATTGAGTTAAGCACATAAAA
Lid_131	GAGAATATAGAGATCTACAAAGGCATTCTACT
Lid_132	GCACCCATAGCATTATAAATTAGGGTAGCT
Lid_133	TCAGACTCACCGTCACCGACTTAAATATTG
Lid_136	CAAAATTATTTTAATGGAAACAGTTAGGTTGG
Lid_137	TTTTTGAAAAATAATATCCCAGATAAGTCCTGAACAATTTTT
Lid_138	TTACCCTTACCAACGCTAACGAGCTATTTT
Lid_139	GTCTGTCAATATCCAGAACAATATTACCGC
Lid_140	AACATGTGAAAAGGTGGCATCATATCAGGT
Lid_141	GCATTTTGACGACAGTATCGGCTCGCACTC
Lid_142	CTGAACCTGTTGGCAAATCAACAGACAATTCG
Lid_143	TTTATTTCAACGCAACAATATGAGGCCGGAGACAGTCAATTTTT
Lid_144	ATGTTACACGTAACAAAGCTGCATTTCAAC
Lid_145	TCAATCAAAAAACAGGAAGATTCTTTCATC
Lid_146	AAGAGCAAAAGCCCTTTTTAAGAAATAAGTTT
Lid_147	AAAGCGAAGGTGAGGCGGTCAGAAAATACC
Lid_148	TTTTTCTGGTAATAAGTTTTAATGATACAGGAGTGTATTTTT
Lid_149	TAGAAAAACTTACCAGTATAAAGCCTGTTTAT
Lid_150	AGCGCGAAATGCCACTACGAAGGCCCACGCAT
Lid_151	ATTTTGTCCGCCAAAGACAAAAGGACCAGTAG
Lid_154	ACACTGAAAGGATTAGGATTAGAACATGAA

Lid_155	AGTAGCGAGAGCCAGCAAAATCGCGACATT
Lid_156	TTTGCGGTATTAGACTTTACAATTGAAAGGAATTGAGGAAAAAT
Lid_157	TTTTTCACTAAAACACTCATCAAGAGGCAAAAGAATATTTTT
Lid_158	GCGGGCCATGTGCTGCAAGGCGCTGTTTCC
Lid_159	GAGCACTAACAGTGCCACGCTGGCGAACTG
Lid_160	TGCTCAGTCCCAATAGGAACCCATGTACCGTA
Lid_161	AAGAACTAACGAGAATGACCATTCAGGTCT
Lid_162	GGCTACAATCTTTTCATAATCAGGCATTTT
Lid_163	CGAAAAACCATCACCCAAATCACCGCTACA
Lid_164	CAGCGGAGGTAACGATCTAAAGTTTTGTCGTC
Lid_165	AGGTTGAGCCACCCTCAGAACCCCGTAATC
Lid_166	GGGCGCGTGCTTTCCTCGTTAGCGTTGTAG
Lid_167	TAGCAAGACCAGGCGGATAAGTCCCCTGCC
Lid_168	TTTTTTTTCTGTATGGGATTTACGTTAGTAAATGAATTTTTT
Lid_169	TTTTTTCATATGGTTTACCAGACAATCAATAGAAAATTTTTT
Lid_170	ATGCAATGGTGAGAAATATTCAACCGTTCTCATACAGGCAAGGCAAAG
Lid_171	CCCTCAGCAGAGGCTGAGATTGAATCACGCTC
Lid_172	AATGGATTTAAAACATCGCCATTATATTAACA
Lid_173	CTCAAGAGGTTTCGTCACCAGTACAAACTACA
Lid_174	CATAAAGCGTAAAGATTCAAAAGGCCTGAGTAA
Lid_175	TTTTTTATAGAAGGCTTATCCTAGCAAGCAAATCAGATTTTT
Lid_176	TGAATTTATATAAACAGTTAATGCGCCGTCGA
Lid_177	CAACAATATCCTAATTTACGAGCATCATTACC
Lid_178	AAAACGATTTGACCCCCAGCGAACCAACTT
Lid_179	GAGGGTTGAGCCACCACCCTCATTTTCAGGGA
Lid_181	TAAATTGACGCAGTATGTTAGCATACCCAA
Lid_182	TTTTTTAATCAGAAAAGCCCCTATGTACCCCGGTTGATTTTT
Lid_183	TGAAAGAAGGCTGGCTGACCTTTCAGGACG
Lid_184	GGCTTTTGACGGGGTCAGTGCCTTGGAATAGGTGTATCACGAACCGCC
Lid_185	TTTTTAATAGCTATCTTACCGGAAACAATGAAATAGCTTTTT
Lid_186	TTTTTACCTGAAAGCGTAAGAAGATAGAACCCTTCTGTTTTT
Lid_187	CAGCCAGATTAAGACGCTGAGATTCCCTTA
Lid_188	TAACCAACGTAACCGTGCATCTCTGGTGCC
Lid_189	TTTTTCGAACGTTATTAATTTTATTAAATCCTTTGCCTTTTT
Lid_190	GTTTACCATACTGCGGAATCGTGGAAGCCC
Lid_191	TTTTTAGCAAGCGGTCCACGCGCCCTGAGAGAGTTGCTTTTT
Lid_192	AACCGATATTATCAGCTTGCTTTCAACAGTTT
Lid_193	AGTTAGCTGAGAATAGAAAGGATTTAATTG
Lid_194	TAAATCGTATTCATTTCAATTATTTGAATA
Lid_195	TTCCAAGCCAGACGACGACAATCGCCATAT
Lid_196	TTTTTACATTATGACCCTGTAATACTTTTGCGGGAGAAGCC
Lid_197	CTAAAGCAATATTTTTGAATGGCCCAGTAATAAAAGGG
Lid_198	TTGCTCCTTTCAAATATCGCGTTTGACTGGAT
Lid_199	TTTTTTAGTTGCGCCGACAATAACAGCTTGATACCGATTTTT

Lid_200	GAAAGACTTTGATAAGAGGTCAAAGTACGG
Lid_201	GCTTCAATCAGGATTAGAGAGTAACAGTTG
Lid_202	CGTAGATTCAGTACCTTTTACATCCAAGAAAA
Lid_203	ACAACTCGTAAAAGTTTGAGTAACAGAAATTG
Lid_204	TTAACAAGACCGTGTGATAAATTTCATCTT
Lid_205	TTTCTTAGACAACAACCATCGCACCAACCT
Lid_206	ATTCCCATAGATACATTTCGCACTAGCATG
Lid_207	CGGCCAAGTTTGGATTATACTTTATCAGAT
Lid_209	TTTTTAACCCGTCGGATTCTCAATGTGAGCGAGTAACTTTTT
Lid_211	TCAGAGCCGGCAGGTCAGACGATTGCAGTCTC
Lid_212	TTTTTTCATTTGAATTACCTTATTACATTTAACAATTTTTTT
Lid_213	TTTTTAAATGAAAATAGCAGCTTTTGTTTAACGTCAATTTTT
Lid_214	TTTTTCAATTCCACACAACATAATTGTTATCCGCTCATTTTT
Lid_216	TATCGGTTATTCGGTCGCTGAGCGGGTAAA
Lid_217	GGAACCAGAAACAAATAAACGAGGCGGAATCC
Lid_218	CAGAGCCAAGAGCCGCCGCCAGCATCATACAT
Lid_219	TTTTTAATCAATATCTGGTCACAAATATCAAACCCTCTTTTT
Lid_220	ACCCTCAGAACCGCCACCCTCAGATATAAGTATAGCCCGAGTAACA
Lid_221	ATACGTAACAAAGTACAACGGAGCAGACGG
Lid_222	GAAGCCTGCACTCATCGAGAACCCTTATCA
Lid_223	TTTTTCACCAGAACCACCACCCCACCCTCAGAGCCGCTTTTT
Lid_224	CAACCGACAAAGACACCACGGAAAGTAAGC
Lid_225	TATTCACAGCCACCACCGGAACCTTTAGCG
Lid_227	CGAGGAATTGAGCGCTAATATCGAATTAAC
Lid_229	CCAAATCATTAGCCGGAACGAGGCGATTTGTA
Lid_210	TAGCTTAGCTTTCCGGCACCGCTTGCCAGTTT
Lid_215	TAGTGAATAAATTTAATGGGAAGACTCAGTTT
Lid_152	GTTCAGAAGGCATGATTAAAAGGAGCCAAGAC
Lid_180	GAGGGTAAACGCAATAATAACGGAAAACGTAG
Lid_065	ATAACCTGAAGTTTCATTCCATATACCTTTAA

Table s7 | Native staple strands of the lids.

Name	Sequence (5' to 3')
Lid_226	AAAGAAGATGTGAGTGAATAACCTGTCTGAGA
Lid_118	TTGTAAAAAGGCAAAGCGCCATTCGTGTAGAT

Table s8 | Additional native staple strands of the "up" lid.

Name	Sequence (5' to 3')
Lid_064	AAGAAACGTTGAGGGAGGGAAGGTGAGCCATT
Lid_007	TTCATTGAAAGAGCAACACTATCAATACCACA

Table s9 | Additional native staple strands of the "down" lid.

Name	Sequence (5' to 3')
Lid_071_SE_up	CACATTAATAGAGGATCCCCGGGTTCACGACGTTTTT**GATTGCGACCAACAGCAG**
Lid_135_SE_up	TTCCAGTCTGAAAACATAGGCATGAGCTTCGATTTTT**GAGTGCCTACTTCGCATC**
Lid_106_SE_up	AGGCGAATTCGCTATTAATTAATTAGAGTCAATTTTT**TCAGACACTGCGACTGCT**

Lid_091_SE_up	**GCTTTTCCGACGCATCCA**TTTGACTACCTATATATTTTAGTTAATAAGGCGTT
Lid_228_SE_up	**TTTGGGTCACATCCAGTG**TTTAAATAAGAGGGCTTAATTGAGAATAAACAACA
Lid_055_SE_up	**AGTGACCCTACCTTACCC**TTTTGTTCAGCATAATCGGCTGTCTTTAAGCAAGC
Lid_061_SE_up	**TTCCACAAACCCGGACAA**TTTCGTTTTTAACCTCCCGACTTGCGGAAAATAAA
Lid_134_SE_up	**CGGGTTAGCCAGAGTAGT**TTTCAGCCATAAGCGCATTAGACGGGAAGAGAGAT
Lid_153_SE_up	**TCAACTCGAAGGCGCACT**TTTAACCCACACGAACAAAGTTACCAGACATATAA
Lid_064_SE_up	**TAGCGCCACGACTAGTAC**TTTTAAGAAACGTTGAGGGAGGGAAGGTGAGCCATT
Lid_093_SE_up	**TTCATCCACTCCACTGAC**TTTTAAAATACATTATTCATTAACCAGAAAACAAGG
Lid_082_SE_up	**CCATGTGTGCACTTGCAT**TTTTTCCTTATTGGCTTGAGATGGTTTATCATTCAG
Lid_052_SE_up	**TAGGCGACGTAAACTCTG**TTTTCAACTAATTGTGAATTACCTTATTCATTAC
Lid_007_SE_up	**TCCCATCGACACGCTTAG**TTTTCATTGAAAGAGCAACACTATCAATACCACA
Lid_122_SE_up	**CGTCTAGTCTGCGTTAGC**TTGATGGCTTATCAAAAAGATTAAGACATAAATA
Lid_060_SE_up	**CCACACTTGGAACGCGTA**TTCGCGAGCTTTTAAATATGCAACTATTTTTGCG
Lid_008_SE_up	**CGCGTATCCACAGTAGTT**TTTTAAAATTTGAGAGTCTGGAGCAAATTTGGGG
Lid_208_SE_up	**GCTATCCGTGACAAGGTC**TTGGGCGCATTAGGAACGCCATCAAAATATTTTG

Table s10 | "Up" lid's staples for hinges conjugations.

Name	Sequence (5' to 3')
Lid_226_SE_D	AAAGAAGATGTGAGTGAATAACCTGTCTGAGATTTT**CCGTACGCTTCACCATAT**
Lid_106_SE_D	AGGCGAATTCGCTATTAATTAATTAGAGTCAATTTT**ACACCAGGGTCGGGTATA**
Lid_135_SE_D	TTCCAGTCTGAAAACATAGGCATGAGCTTCGATTTT**TGTAAGAGAGGGCATCGC**
Lid_071_SE_D	CACATTAATAGAGGATCCCCGGGTTCACGACGTT**AGGAGCCGACATCCTCTT**
Lid_118_SE_D	TTGTAAAAAGGCAAAGCGCCATTCGTGTAGATTT**CTTAGCAGTGCTTGGATC**
Lid_208_SE_D	GGGCGCATTAGGAACGCCATCAAAATATTTTGTT**ACTGTGTGCTCTAGGGAT**
Lid_008_SE_D	TTAAAATTTGAGAGTCTGGAGCAAATTTGGGGTT**ACTGTACTAGGTGGTCGC**
Lid_060_SE_D	CGCGAGCTTTTAAATATGCAACTATTTTTGCGTT**AATGGGCGGAGAGTTCAG**
Lid_122_SE_D	GATGGCTTATCAAAAAGATTAAGACATAAATATT**AACCTCTACGCCTACCTG**
Lid_052_SE_D	CTAGGACCCAGATTGTTGTTTTTTTCAACTAATTGTGAATTACCTTATTCATTAC
Lid_082_SE_D	CCGAGATCGGATCCTACATTTTTTCCTTATTGGCTTGAGATGGTTTATCATTCAG
Lid_093_SE_D	TCTGTGTTGTGCATACGCTTTTTAAAATACATTATTCATTAACCAGAAAACAAGG
Lid_153_SE_D	AACCCACACGAACAAAGTTACCAGACATATAATTT**TGGCATAGCTTCCGTCCT**
Lid_134_SE_D	CAGCCATAAGCGCATTAGACGGGAAGAGAGATTTT**TCAGGATCAAGCACAGGT**
Lid_061_SE_D	CGTTTTTAACCTCCCGACTTGCGGAAAATAAATTT**CACTGTCAGGCCATCACT**
Lid_055_SE_D	TGTTCAGCATAATCGGCTGTCTTTAAGCAAGCTTT**CCTTGTGCCTAGTGCCAT**
Lid_228_SE_D	AAATAAGAGGGCTTAATTGAGAATAAACAACATTT**TGCCTCACACACCGGTTA**
Lid_091_SE_D	GACTACCTATATATTTTAGTTAATAAGGCGTTTTTT**TCAAATCCCGGCTTGTCC**

Table s11 | "Down" lid's staples for hinges conjugations.

Name	Sequence (5' to 3')
Lid_071	CACATTAATAGAGGATCCCCGGGTTCACGACG
Lid_135	TTCCAGTCTGAAAACATAGGCATGAGCTTCGA
Lid_106	AGGCGAATTCGCTATTAATTAATTAGAGTCAA
Lid_064	AAGAAACGTTGAGGGAGGGAAGGTGAGCCATT
Lid_093	AAAATACATTATTCATTAACCAGAAAACAAGG

Lid_082	TCCTTATTGGCTTGAGATGGTTTATCATTCAG
Lid_052	TTCAACTAATTGTGAATTACCTTATTCATTAC
Lid_007	TTCATTGAAAGAGCAACACTATCAATACCACA
Lid_122	GATGGCTTATCAAAAAGATTAAGACATAAATA
Lid_060	CGCGAGCTTTTAAATATGCAACTATTTTTGCG
Lid_008	TTAAAATTTGAGAGTCTGGAGCAAATTTGGGG
Lid_208	GGGCGCATTAGGAACGCCATCAAAATATTTTG

Table s12| "Up" lid's staples for no hinge's conjugations.

Name	Sequence (5' to 3')
Lid_226	AAAGAAGATGTGAGTGAATAACCTGTCTGAGA
Lid_106	AGGCGAATTCGCTATTAATTAATTAGAGTCAA
Lid_135	TTCCAGTCTGAAAACATAGGCATGAGCTTCGA
Lid_071	CACATTAATAGAGGATCCCCGGGTTCACGACG
Lid_118	TTGTAAAAAGGCAAAGCGCCATTCGTGTAGAT
Lid_208	GGGCGCATTAGGAACGCCATCAAAATATTTTG
Lid_008	TTAAAATTTGAGAGTCTGGAGCAAATTTGGGG
Lid_060	CGCGAGCTTTTAAATATGCAACTATTTTTGCG
Lid_122	GATGGCTTATCAAAAAGATTAAGACATAAATA
Lid_052	TTCAACTAATTGTGAATTACCTTATTCATTAC
Lid_082	TCCTTATTGGCTTGAGATGGTTTATCATTCAG
Lid_093	AAAATACATTATTCATTAACCAGAAAACAAGG

Table s13 | "Down" lid's staples for no hinge's conjugations.

Name	Sequence (5' to 3')
Lid060_vavKclose	AGGTCATCGCTGAGGGGCAAGCTGATTTCGCGAGCTTTTAAATATGCAACTATTTTTGCG
Lid065_vavKfar	AGGTCATCGCTGAGGGGCAAGCTGATTTATAACCTGAAGTTTCATTCCATATACCTTTAA

Table s14| Staples for spring conjugations – both lids.

REFERENCES

1. Douglas, S. M. *et al.* Rapid prototyping of 3D DNA-origami shapes with caDNAno. *Nucleic Acids Res.* **37**, 5001–5006 (2009).
2. Lee, J. Y. *et al.* Rapid Computational Analysis of DNA Origami Assemblies at Near-Atomic Resolution. *ACS Nano* **15**, 1002–1015 (2021).
3. Rothemund, P. W. K. Folding DNA to create nanoscale shapes and patterns. *Nature* **440**, 297–302 (2006).
4. Wu, H. *et al.* Expanding DNA Origami Design Freedom with De Novo Synthesized Scaffolds. *J. Am. Chem. Soc.* **146**, 16076–16084 (2024).
5. Landry, M. P., McCall, P. M., Qi, Z. & Chemla, Y. R. Characterization of Photoactivated Singlet Oxygen Damage in Single-Molecule Optical Trap Experiments. *Biophys. J.* **97**, 2128–2136 (2009).
